# Enhancing Production of Pinene in *Escherichia coli* by Using a Combination of Tolerance, Evolution, and Modular Co-culture Engineering

**DOI:** 10.3389/fmicb.2018.01623

**Published:** 2018-07-31

**Authors:** Fu-Xing Niu, Xin He, Ya-Qin Wu, Jian-Zhong Liu

**Affiliations:** Institute of Synthetic Biology, Biomedical Center, Guangdong Province Key Laboratory of Improved Variety Reproduction in Aquatic Economic Animals and South China Sea Bio-Resource Exploitation and Utilization Collaborative Innovation Center, School of Life Sciences, Sun Yat-sen University, Guangzhou, China

**Keywords:** pinene biosynthesis, *Escherichia coli*, tolerance engineering, directed evolution, chemically induced chromosomal evolution, modular co-culture

## Abstract

α-Pinene is a natural and active monoterpene, which is widely used as a flavoring agent and in fragrances, pharmaceuticals, and biofuels. Although it has been successfully produced by genetically engineered microorganisms, the production level of pinene is much lower than that of hemiterpene (isoprene) and sesquiterpenes (farnesene) to date. We first improved pinene tolerance to 2.0% and pinene production by adaptive laboratory evolution after atmospheric and room temperature plasma (ARTP) mutagenesis and overexpression of the efflux pump to obtain the pinene tolerant strain *Escherichia coli* YZFP, which is resistant to fosmidomycin. Through error-prone PCR and DNA shuffling, we isolated an *Abies grandis* geranyl pyrophosphate synthase variant that outperformed the wild-type enzyme. To balance the expression of multiple genes, a tunable intergenic region (TIGR) was inserted between *A. grandis GPPS*^*D*90*G*/*L*175*P*^ and *Pinus taeda Pt1*^*Q457L*^. In an effort to improve the production, an *E. coli-E. coli* modular co-culture system was engineered to modularize the heterologous mevalonate (MEV) pathway and the TIGR-mediated gene cluster of *A. grandis GPPS*^*D*90*G*/*L*175*P*^ and *P. taeda Pt1*^*Q457L*^. Specifically, the MEV pathway and the TIGR-mediated gene cluster were integrated into the chromosome of the pinene tolerance strain *E. coli* YZFP and then evolved to a higher gene copy number by chemically induced chromosomal evolution, respectively. The best *E. coli-E. coli* co-culture system of fermentation was found to improve pinene production by 1.9-fold compared to the mono-culture approach. The *E. coli-E. coli* modular co-culture system of whole-cell biocatalysis further improved pinene production to 166.5 mg/L.

## Introduction

α-Pinene is a natural and active monoterpene, which is widely used in flavorings, fragrances, insecticides, pharmaceuticals, and fine chemicals (Breitmaier, 2006; Behr and Johnen, 2009; Kirby and Keasling, 2009; Gandini and Lacerda, 2015). It was recently produced as a candidate renewable jet fuel due to its favorable energy content, cold weather properties, and high octane/cetane numbers (George et al., 2015). The main source of pinene is turpentine, a by-product of the wood pulp industry (Behr and Johnen, 2009). However, this extraction from plants is tedious and inefficient and requires substantial expenditure of natural resources due to low content (Chang and Keasling, 2006). Therefore, there is much interest in developing biotechnologies for pinene production from renewable resources by engineering microorganisms. Similar to other monoterpenes, α-pinenes are biosynthesized from the C5 intermediates isopentenyl diphosphate (IPP) and dimethylallyl diphosphate (DMAPP) via geranyl diphosphate synthase (GPPS). The head-to-tail condensation produces geranyl diphosphate (GPP, C10), which is, in turn, cyclized by pinene synthase (PS) to produce either α- or β-pinene. *Escherichia coli* (Yang et al., 2013; Sarria et al., 2014; Tashiro et al., 2016) and *Corynebacterium glutamicum* (Kang et al., 2014) have been engineered to produce α-pinene. α-Pinene (5.4 mg/L) has been produced in engineered *E. coli* through the introduction of a heterologous mevalonate (MEV) pathway and α-pinene synthase (Pt30) from *Pinus taeda* (Yang et al., 2013). The combinatorial expression of *Abies grandis* GGPS-PS fusion proteins enhanced pinene production (32 mg/L) in *E. coli* (Sarria et al., 2014). The directed evolution of α-pinene synthase (Pt1) from *P. taeda* increased α-pinene productivity. *E. coli* plasmid-expressing the evolved α-pinene synthase (Pt1^Q457L^) from *P. taeda*, MEV pathway enzymes, IPP isomerase and *A. grandis* GGPS produced the highest levels of pinene (140 mg/L) in a flask culture to date (Tashiro et al., 2016). The coexpression of native 1-deoxy-d-xylulose-5-phosphate synthase (Dxs) and isopentenyl diphosphate isomerase (Idi) with *P. taeda* PS and *A. grandis* GPPS in *C. glutamicum* yielded a pinene level of 27 μg/g cell dry weight (Kang et al., 2014).

However, the production level of pinene is much lower than that of hemiterpene (isoprene) (Whited et al., 2010) and sesquiterpenes (farnesene) (Zhu et al., 2014) to date. Pinene is highly toxic to *E. coli*. *E. coli* growth is inhibited by 0.5% pinene (Dunlop et al., 2011). The inherent tolerance of *E. coli* may limit the production potential. It was demonstrated that increasing the tolerance of *E. coli* by overexpressing the efflux pump AcrBDFa (YP_692684) from *Alcanivorax borkumensis* significantly enhanced limonene production (Dunlop et al., 2011). Another reason for the lower yield may be that PS has a lower expression level and/or lower enzymatic activity in *E. coli*. Thus, we first combined tolerance engineering with directed evolution of the enzyme to improve pinene production in *E. coli*.

Recently, there has emerged a new modular co-culture engineering approach for engineering microorganisms. Modular co-culture engineering approaches divide a complete biosynthetic pathway into separate serial modules, which are introduced into different strains to accommodate individual modules for achieving designed biosynthesis (Zhang and Wang, 2016). The advantages of using modular co-culture engineering include the following: (1) reducing the metabolic burden on each host strain; (2) providing diversified cellular environments for functional expression of the different pathway genes; (3) reducing the undesired interference of different pathways; (4) easily balancing the biosynthetic pathway between individual pathway modules by simply changing the strain-to-strain ratio; (5) high-efficiency utilization of complex materials containing multiple active substrates; and (6) supporting the plug-and-play biosynthesis of various target products (Zhang and Wang, 2016). Thus, modular co-culture engineering was also used to enhance pinene production in *E. coli*.

## Materials and methods

### Strains, plasmids, and primers

The bacterial strains and plasmids used in this study are listed in Table 1. The primers used in this study are listed in Supplementary Table 1.

**Table 1:** Strains and plasmids used in this study.

**Name**	**Description**	**Reference/Sources**
**STRAIN**		
*E. coli* BW25113	*lacI*^q^*rrnB*_T14_Δ*lacZ*_WJ16_*hsdR514 ΔaraBAD*_AH33_Δ*rhaBAD*_LD78_	Datsenko and Wanner, 2000
*E. coli* BW25113 (P_T5_-dxs)	*E. coli* BW25113, P_dxs_::P_T5_	Weng et al., 2012
*E. coli* YZ-3	The ALE strain from *E. coli* BW25113 (P_T5_-dxs), tolerance to 2.0% pinene	This study
*E. coli* YZ-3-A	*E. coli* YZ-3, P_acrAB_::P37	This study
*E. coli* YZ-3-A-T	*Pseudomonas putida* KT2440 *ttgB* under the control of P37 promoter was integrated into the chromosome of *E. coli* YZ-3-A	This study
*E. coli* YZFP	Pinene tolerance strain, *E. coli* YZ-3-A-T mutant resistant to fosmidomycin	This study
*E. coli* PINE	Pinene producer, CIChE strain from *E. coli* YZFP after integration of the TIGR-mediated gene cluster of the *A. grandis* GPPS^Mut^-*P. taeda* Pt1^MUT^ gene cluster	This study
*E. coli* MEVI	CIChE strain from *E. coli* YZFP after integration of the mevalonate pathway	This study
**PLASMID**		
pJBEI-6409	Addgene plasmid #47048, pBbA5c-MTSAe-T1f-MBI(f)-T1002i-Ptrc-trGPPS(co)-LS) coding for MEV pathway enzymes to produce limonene from glucose in *E. coli*, p15A *ori*, P_lacUV5_ promoter, cm^r^	Alonso-Gutierrez et al., 2013
pMEVI	pBbA5c-MTSAe-T1f-MBI(f)-T1002i coding for MEV pathway enzymes and *E. coli* Idi, p15A *ori*, P_lacUV5_ promoter, cm^r^	This study
pMEVIGPS	pBbA5c-MTSAe-T1f-MBI(f)-T1002i-trGPPS*_*A*.*grandis*_*-PS*_*A*.*grandi*_* coding for MEV pathway enzymes to produce pinene from glucose in *E. coli*, p15A *ori*, P_lacUV5_ promoter, cm^r^	This study
pBbA5K-EPL11	Addgene plasmid #45403, pBbA5K containing *Pseudomonas putida* KT2440 *ttgB*	Dunlop et al., 2011
pBbA5K-EPL14	Addgene plasmid #45405, pBbA5K containing *P. putida* KT2440 *mexF*	Dunlop et al., 2011
pBbA5K-EPL95	Addgene plasmid #45434, pBbA5K containing *Alcanivorax borkumensis acrBDFa*	Dunlop et al., 2011
pZEABP	Constitute expression vector, pBR322 *ori*, P37 promoter, Amp^r^, BglBrick, ePathBrick containing four isocaudamer (AvrII, NheI, SpeI, and XbaI)	Li et al., 2015
pZEA-acrB	pZEA^*^BP containing *E. coli acrB*, pBR322 *ori*, P37 promoter, Amp^r^	This study
pZEA-acrAB	pZEA^*^BP containing *E. coli acrAB*, pBR322 *ori*, P37 promoter, Amp^r^	This study
pZEA-mexF	pZEA^*^BP containing *P. putida* KT2440 *mexF*, pBR322 *ori*, P37 promoter, Amp^r^	This study
pZEA-acrBDFa	pZEA^*^BP containing *A. borkumensis acrBDFa*, pBR322 *ori*, P37 promoter, Amp^r^	This study
pZEA-ttgB	pZEA^*^BP containing *P. putida* KT2440 *ttgB*, pBR322 *ori*, P37 promoter, Amp^r^	This study
pQE30	*E. coli* expression vector, T5 promoter, pBR322 *ori*, Am^r^	Invitrogen
pQE-GPPS-L-PS	pQE30 harboring the fusion gene of the codon-optimized *A. grandis* GPPS and PS with a (GSG)_2_ linker	This study
pQE-GPPS_6AA_-L-PS	pQE30 harboring the fusion gene of the 6AA method optimized *A. grandis* GPPS and PS with a (GSG)_2_ linker	This study
pQE-GPPS-L-PS^epPCR^	pQE30 harboring the evolved fusion gene of the 6AA method optimized *A. grandis* GPPS and PS with a (GSG)_2_ linker after error-prone PCR	This study
pQE-GPPS-L-PS^DNAshuffling^	pQE30 harboring the evolved fusion gene of the 6AA method optimized *A. grandis* GPPS and PS with a (GSG)_2_ linker after error-prone PCR and DNA shuffling	This study
pQE-GPPS^MUT^-L-Pt1^Q457L^	pQE30 harboring the fusion gene of the evolved *A. grandis* GPPS and *P. taeda* Pt1^Q457L^ with a (GSG)_2_ linker	This study
pQE-GPPS^MUT^-Pt1 ^Q457L^	pQE30 harboring *A. grandis* GPPS^D90G/L175P^ and *P. taeda* Pt1^Q457L^	This study
pQE-GPPS^MUT^-TIGR-Pt1^Q457L^	pQE30 harboring the TIGR-mediated gene cluster of the evolved *A. grandis* GPPS and *P. taeda* Pt1^Q457L^	This study
pP_rstA_-GFP	the IPP/FPP sensor plasmid, pZSBP derivative with GFP, P_rstA_ promoter, kan^r^	Shen et al., 2016
pP21KF3T5b	CIChE integration expression vector, *attP*_P21_ site, kan^r^	Chen et al., 2013
pHKKF3T5b	CIChE integration expression vector, *attP*_HK_ site, kan^r^	Chen et al., 2013
pHKKF3T5b-GPPS^MUT^-TIGR-Pt1^Q457L^	pHKKF3T5b harboring the TIGR-mediated gene cluster of the evolved *A. grandis* GPPS and *P. taeda* Pt1^Q457L^	This study
pP21KF3T5b-MEVI	pP21KF3T5b harboring MEV pathway enzymes and *E. coli* Idi	This study
pCas	*E. coli* cas9 expression vector	Jiang et al., 2015
pCas^*^	*E. coli* cas9 (K848A/K1003A/ R1060A) expression vector	This study
pTargetF	*E. coli* sgRNA expression vector	Jiang et al., 2015
pTargetB	*E. coli* sgRNA expression vector, BglBrick vector	This study

### Genetic methods

pMEVI was derived from pJBEI-6409 (Alonso-Gutierrez et al., 2013), which was obtained from Addgene. pJBEI-6409 contains six genes of the MEV pathway (*atoB* from *E. coli, HMGS*, and *HMGR* from *Staphylococcus aureus* and *MK, PMK*, and *PMD* from *Saccharomyces cerevisiae, idi* from *E. coli, GPPS* from *A. grandis*, and limonene synthase gene (*LS*) from *Mentha spicata*). The *GPPS-LS* gene cluster was removed from pJBEI-6409 to obtain pMEVI. The fusion gene cluster of the codon-optimized *GPPS* and *PS* from *A. grandis* with a (GSG)_2_ linker was synthesized by Suzhou GENEWIZ, Inc. (Suzhou, China) and ligated into pQE30 to obtain pQE-GPPS-L-PS. The GPPS-PS gene cluster from pQE-GPPS-L-PS was inserted into the BamHI/XhoI sites of pMEVI to obtain pMEVIGPS. The evolved codon-optimized *Pt1* (*Pt1*^*Q457L*^) from *P. taeda* was synthesized by Suzhou GENEWIZ, Inc. (Suzhou, China) and ligated into pQE30 to obtain pQE-Pt1^Q457L^. The *PS* gene of pQE-GPPS-L-PS^DNAshuffling^ was replaced with the *Pt1*^*Q457L*^ gene to obtain pQE-GPPS^MUT^-L-Pt1^Q457L^.

The *acrB* and *acrAB* were amplified from *E. coli* and inserted into pZEABP to obtain pZEA-acrB and pZEA-acrAB, respectively. The *P. putida* KT2440 *ttgB, P. putida* KT244 *mexF*, and *A. borkumensis acrBDFa* were amplified from pBbA5K-EPL11, pBbA5K-EPL14, and pBbA5K-EPL95 and inserted into pZEABP to obtain pZEA-ttgB, pZEA-mexF, and pZEA-acrBDFa, respectively.

The TIGR-mediated GPPS^MUT^-Pt1^Q457L^ gene cluster was cut from pQE-GPPS^MUT^-TIGR-Pt1^Q457L^ with EcoRI/HindIII and then cloned into EcoRI/HindIII-digested pHKKF3T5b to obtain pHKKF3T5b-GPPS^MUT^-TIGR-Pt1^Q457L^. The MEVI operon was cut from pMEVI with EcoRI/XhoI and then cloned into EcoRI/SalI-digested pP21KF3T5b to obtain pP21KF3T5b-MEVI. Chromosomal integration was carried out by direct transformation as described by Chen et al. (2013). Chemically induced chromosomal evolution (CIChE) of the above construct was carried out by subculturing the resulting strains in 5 mL Super Optimal Broth (SOB) medium with increasing concentrations of triclosan in 15 mL culture tubes, as described by Chen et al. (2013). The strains were grown to the stationary phase in 1 μM triclosan for pP21KF3T5b-GPPS^MUT^-TIGR-Pt1^Q457L^ or 0.25 μM for pHKKF3T5b-MEVI. Fifty milliliters of the culture were subcultured into a new culture tube, in which the triclosan concentration was doubled to 132 or 32 μM and allowed to grow to the stationary phase. The process was repeated until the desired concentration was reached. The *recA* gene of the CIChE strain was then deleted by the markerless deletion approach using the isopropyl β-D-1-thiogalactopyranoside (IPTG)-inducible *ccdB* as a counter-selectable marker (Wei et al., 2016).

Gene replacement of the native promoter of *E. coli acrAB* and the integration of *ttgB* from *P. putida* KT2440 were carried out by the CRISPR-Cas method as described by Jiang et al. (2015). To enhance specificity and reduce off-target effects, the *cas9* on pCas (Jiang et al., 2015) was site-directed mutated into *cas9(K848A/K1003A/R1060A)* as described as Slaymaker et al. (2016) to obtain pCas^*^. To easily assemble the sgRNA sequence using the BglBrick standard method, the BglII site in the sgRNA plasmid pTargetF was first removed, and then a BglII site was added in the front of EcoRI site to obtain the sgRNA plasmid pTargetB.

### Adaptive laboratory evolution for improving pinene tolerance

A 1-mL culture of logarithmic phase *E. coli* was collected by centrifugation, washed twice with saline, and diluted to a cell concentration of 10^6^ to 10^7^ with physiological saline. Then, atmospheric and room temperature plasma (ARTP) mutagenesis was performed using an ARTP mutation system (ARTP-IIS, Tmaxtree Biotechnology Co, Ltd, Wuxi, China) with the following parameters: (1) the radio frequency power input was 100 W; (2) the flow of pure helium was 10 standard liters per min; (3) the distance between the plasma torch nozzle exit and the slide was 2 mm; and (4) the different treatment times were selected (10, 20, 40, 60, 80, 100, and 120 s). Ten microliters of the aforementioned cell dilution were evenly scattered on the slide and subjected to ARTP mutagenesis. After treatment, the slide was washed with LB medium (10 g/L tryptone, 5 g/L yeast extract, 10 g/L NaCl), transferred to 5 mL of LB medium with 0.5% pinene in a 15 mL falcon tube, and cultivated at 30 °C and 200 rpm for 24 h. The cultures were serially passed into fresh medium (initial OD_600_ of 0.2) daily. Continuously repeating this transfer procedure at 0.5% pinene until OD_600_ at 24 h did not increase further, the culture was then sequentially transferred to a pinene concentration of 1.0%, 1.5% and 2.0%. The cultures were frozen and stored at −80°C at every pinene concentration.

The cultures of 2.0% pinene stored at −80°C were transferred by the IPP/FPP sensor plasmid pP_rstA_-GFP. Single colonies were inoculated in individual wells of a 48 deep-well microplate (4.6 mL) containing 600 μL of LB medium and incubated at 30°C and 200 rpm for 24 h on a Multitron shaker (Infors). The cells were harvested by centrifugation at 14000 × g for 2 min and then resuspended with 0.6 mL (137 mM NaCl, 2.7 mM KCl, 10 mM Na_2_HPO_4_, 2 mM KH_2_PO_4_, pH 7.4). Then, 200 μL of the bacterial culture was transferred into a 96-well plate in which the OD_600_ and fluorescence were read with the excitation at 485 nm and emission at 528 nm using a SynergyNeo2 multi-mode reader (SynergyNeo2, BioTek, USA).

### Generating random mutagenesis libraries using error-prone PCR and screening

The random mutagenesis libraries of the fusion gene cluster of *AgGPPS-AgPS* after optimization of the first 18 codons using the 6AA method (Boë et al., 2016) were constructed through error-prone PCR. The gene cluster of *AgGPPS-AgPS* was amplified from pQE-GPPS_6AA_-L-PS using the primers EcoRI-GPPS/HindIII-PS. The error-prone PCR reaction mixture consisted of 5 mM MgCl_2_, 0.3 mM MnCl_2_, 0.2 mM each of dATP and dGTP, 1 mM each of dCTP and dTTP and Tag DNA polymerase. The PCR product was digested by EcoRI/HindIII, ligated into the EcoRI/HindIII sites of pQE30, and then transferred into the lycopene-producing strain *E. coli* LYCOP to generate the mutant library.

The mutant library was plated on LB agar with ampicillin and IPTG. The plates were incubated at 30°C overnight. The mutant plasmid was isolated from the whiter colony and then transferred into component *E. coli* BW25113 (P_T5_-dxs, pMEVI). The pinene productions of them were analyzed in a shake flask.

### Generating random mutagenesis libraries using DNA shuffling and screening

DNA shuffling experiments were performed by the following steps: parental template preparation, DNase I digestion, primer-less PCR and PCR with primers. The mutant plasmids from the 7 colonies resulted from error-prone PCR and were used as the template to amplify the gene cluster fragments with the primers EcoRI-GPPS/HindIII-PS. Following purification, 2 μg of the eight PCR products was mixed and treated with 0.02 U of DNaseI in 100 μL of the 10 × DNaseI buffer on ice for 2 min and terminated by the loading buffer containing SDS. The purified fragments of 50–300 bp were used in the primer-less PCR reactions to reassemble into full-length genes. The primer-less PCR reaction mixture contained 0.5 mM each dNTP, 10 × Taq buffer and 0.5 μL Taq DNA polymerase (Takara). The PCR reaction conditions were as follows: 95°C for 1 min, 35 cycles of 94°C for 30 s, 45°C for 30 s, 72°C for 3 min, and final incubation at 72°C for 8 min. The PCR products with the correct size were purified and subjected to PCR amplification using the same conditions with the primers EcoRI-GPPS/HindIII-PS. Finally, the mutated PCR products of the full-length gene were digested by EcoRI/HindIII, ligated into the EcoRI/HindIII sites of pQE30, and transferred into the lycopene-producing strain *E. coli* LYCOP to generate the mutant library.

The mutant library was plated on LB agar with ampicillin and IPTG. The plates were incubated at 30°C overnight. Single colonies with a whiter color were inoculated in individual wells of a 48 deep-well microplate (4.6 mL) containing 1 mL of LB medium and incubated at 30°C and 200 rpm on a Multitron shaker (Infors). After 8 h, the cultures were induced with 1 mM IPTG and overlaid with 20% dodecane to trap pinene. After induction, the cultures were incubated at 30°C and 200 rpm for 48 h. The pinene concentration in individual wells was assayed using the concentrated sulfuric acid method as follows. One hundred microliters of the dodecane layer were mixed with 200 μL sulfuric acid, then inoculated for 5 min in boiling water, and the absorbance of the reaction solution at 450 nm was determined using a spectrophotometer (Shimadzu, Japan).

### Creating TIGR libraries and screening

TIGRs were synthesized using PCR to assemble the oligonucleotides into chimeric DNA sequences as described by Pfleger et al. (2006) and Li et al. (2015). Briefly, 40 mmols of an equimolar oligonucleotide (A, B, C, and D in Supplemental Table 1) mixture was added to a mixture containing 2.5 units of Primer Star DNA Polymerase (Takara, Dalian, China). The assembly was conducted over 35 cycles of PCR for 10 s at 98°C, 30 s at 72°C, and 20 + 5 s/cycle at 72°C. The assembly products were purified using a nucleotide removal column and amplified using the end-specific primers TIGRs-F(X)/TIGRs-R(A) and then cloned into the SacI/SalI sites of pQE-GPPS^MUT^-Pt1 ^Q457L^ to obtain the plasmid libraries pQE-GPPS^MUT^-TIGRs-Pt1^Q457L^. The plasmid libraries were transferred into component *E. coli* BW25113 (P_T5_-dxs, pMEVI) to generate the mutant library.

The TIGR library was plated on LB agar with ampicillin. The plates were incubated at 30°C overnight. Single colonies were inoculated in individual wells of a 48 deep-well microplate (4.6 mL) containing 1 mL of LB medium and incubated at 30°C and 200 rpm on a Multitron shaker (Infors). After 8 h, the cultures were induced with 1 mM IPTG and overlaid with 20% dodecane to trap pinene. After induction, the cultures were incubated at 30°C and 200 rpm for 48 h. The pinene concentration in individual wells was assayed using the above concentrated sulfuric acid method.

### Pinene biosynthesis in shake flasks

For pinene fermentation production, a single colony was inoculated into 5 mL of LB medium in a falcon tube, which was cultured overnight at 37°C. The overnight seed culture was then inoculated into 50 mL of SBMSN medium with a starting OD_600_ of 0.1. SBMSN medium (pH 7.0) containing the following (g/L): sucrose 20, peptone 12, yeast extract 24, KH_2_PO_4_ 1.7, K_2_HPO_4_ 211.42, MgCl_2_·6H_2_O 1, ammonium oxalate 1.42, and Tween-80 2. The main cultures were then incubated at 37°C and 200 rpm until an OD_600_ of 0.8 was reached. Then, the cultures were induced with 1 mM IPTG and overlaid with 20% dodecane to trap pinene. After induction, the cultures were incubated at 30°C and 130 rpm for 72 h.

### Co-culture of *E. coli* PINE and MEVI for pinene production

*E. coli* PINE and MEVI cells were first separately grown in 5 mL SBMSN medium in a falcon tube at 37°C overnight. The overnight culture was inoculated into 50 mL of SBMSN medium with a starting OD_600_ of 0.1 and incubated at 37°C and 200 rpm until an OD_600_ of approximately 6.0 was reached. The cultures were then incubated at 20°C and 200 rpm for 16 h. For pinene biosynthesis using co-cultures, the *E. coli* PINE culture and the desired amount of the *E. coli* MEVI culture were inoculated into the 30 mL SBMSN medium with a starting OD_600_ of 0.1. The mixed culture was culture at 37°C and 200 rpm until an OD_600_ of 0.8 was reached. Then, the cultures were overlaid with 20% dodecane to trap pinene, and were incubated at 30°C and 130 rpm for 72 h.

### Whole-cell biocatalysis for pinene production

A single colony of *E. coli* PINE and MEVI was separately inoculated into 5 mL of SBMSN medium in a falcon tube, which was cultured overnight at 37°C. The overnight cultures were then inoculated into 50 mL SBMSN medium with a starting OD_600_ of 0.1. The cultures were then incubated at 37°C and 200 rpm until an OD_600_ of approximately 6.0 was reached. Then, the cultures were incubated at 20°C and 200 rpm for 16 h. Finally, the *E. coli* PINE culture was mixed with the *E. coli* MEVI culture at the inoculation ratio of 2:1. The mixed cells were harvested by centrifugation (6000 × g at 4°C) and washed twice with cooled phosphate buffer (0.1 M, pH 7.0).

For biocatalysis, the above cells were resuspended in 10 mL phosphate buffer (0.1 M, pH 7.0) containing 20 g/L of sucrose, 10 mM MgCl_2_ and 5 mM MnCl_2_ to form the cell suspension (OD_600_ = 30). The reaction mixture was overlaid with 20% dodecane. The catalysis was performed for 28 h at 30°C and 130 rpm.

### GC analysis

Five hundred microliters of the dodecane layer was placed in a 1.5-mL microcentrifuge tube and centrifuged at 25,000 × g for 1 min, and 50 μL of dodecane was diluted in 450 μL of ethyl acetate spiked with the internal standard limonene (10 μg/L). The samples were analyzed by GC-FID by using a standard curve of α-pinene (Sigma Aldrich). The GC-FID (Techcomp GC7900, Techcomp Ltd, China) was used with a TM-5 column (30 m × 0.32 mm × 0.50 μm). The inlet temperature was set to 300°C, with the flow at 1 mL/min, the oven at 50°C for 30 s, ramp at 4°C/min to 70°C, and ramp at 25°C/min to 240°C.

### Quantitative real-time PCR (qRT-PCR)

The total RNA from *E. coli* cells grown for 24 h in shake flasks was isolated using an RNA extraction kit (Dongsheng Biotech, Guangzhou, China), following the manufacturer's instructions. The first-strand cDNA was synthesized using an All-in-One™ First-Strand cDNA Synthesis kit (GeneCopoeia, Guangzhou, China). The qRT-PCR was perfor1med with the All-in-One™ qPCR Mix kit (GeneCopoeia) on an iCycler iQ5 Real Time PCR system (Bio-Rad Laboratories, California, USA). The template was 100 ng of cDNA. The PCR conditions were as follows: 95°C for 10 min, followed by 45 cycles of denaturation at 95°C for 10 s, annealing at 60°C for 20 s, and extension at 72°C for 15 s. The primers for qRT-PCR are presented in Supplementary Table 1. The data were analyzed by the 2^−ΔΔ*Ct*^ method described by Livak and Schmittgen (2001) and normalized by *cysG* gene expression.

Gene copy numbers were measured by qPCR on genomic DNA isolated from the appropriate CIChE strains. qPCR was performed as described above. The primers QPt1F/QPt1R and QHF/QHR (Supplementary Table 1) were used to measure the copy number of *Pt1* and *HMGS*, respectively.

### Statistical analysis

All experiments were conducted in triplicate, and the data were averaged and presented as the means ± standard deviation. One-way analysis of variance followed by Tukey's test was used to determine significant differences using the OriginPro (version 7.5) package. Statistical significance was defined as *p* < 0.05.

## Results

### Tolerance engineering to improve pinene production

**Figure 1:**
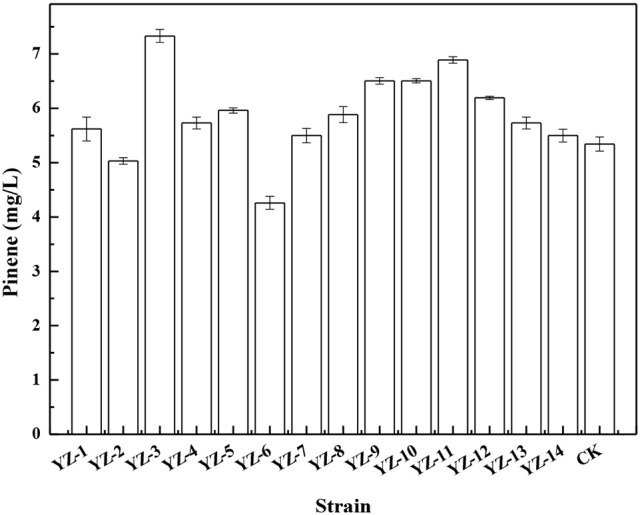
Pinene production by the selected adaptive laboratory evolution strains harboring pMEVIGPS. *E. coli* BW25113 (P_T5_-dxs, pMEVIGPS) was set as the control strain (CK). The data represent the means of three replicates and error bars represent standard deviations.

To improve pinene tolerance, *E. coli* cells harboring pP_rstA_-GFP were treated with ARTP and then serially transferred into LB medium supplemented with increased concentrations of pinene of 0.5, 1.0, 1.5, and 2.0%. The culture was transferred daily. After the adaptive evolution at 2.0% pinene, the culture was streaked on LB plates for isolated colonies. It has been demonstrated that the IPP/FPP sensor plasmid pP_rstA_-GFP has been successfully used to test the intracellular IPP/FPP concentration and to screen the library with higher IPP/FPP concentrations (Dahl et al., 2013; Shen et al., 2016). Thus, we also used it to screen the library. Of the 670 clones, 14 strains with higher fluorescence strength (Supplementary Figure 1) were selected for further shake flask analysis. As shown in Figure 1, *E. coli* YZ-3 produced the highest level of pinene (7.3 ± 0.2 mg/L), which was 31% higher than the starting strain *E. coli* BW25113 (P_T5_-dxs).

**Figure 2:**
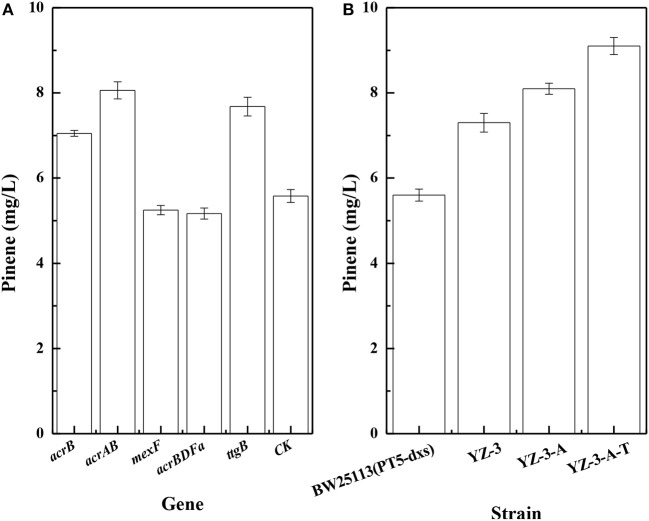
Effect of overexpression of efflux pumps on pinene production. **(A)** Plasmid-expression in *E. coli* YZ-3 (pMEVIGPS). *E. coli* YZ-3 (pMEVIGPS, pZEABP) was set as the control strain (CK); **(B)** Chromosomal-expression in *E. coli* harboring pMEVIGPS. The data represent the means of three replicates and error bars represent standard deviations.

To improve pinene production, we investigated effects of efflux pumps on pinene production. Dunlop et al. (2011) reported that expressing some efflux pumps significantly improved pinene tolerance. Thus, we tested whether pumps that improved pinene tolerance also enhanced its production. As shown in Figure 2A, expressing native AcrB, AcrAB, or TtgB (NP_743544) from *Pseudomonas putida* KT2440 in *E. coli* YZ-3 using plasmid resulted in increased pinene production. However, expressing *A. borkumensis* AcrBDFa (YP_692684) or *P. putida* KT2440 MexF (NP_745564) from did not improve pinene production. Therefore, we first replaced the native promoter of *E. coli* YZ-3 *acrAB* operon with the strong P37 promoter to obtain *E. coli* YZ-3-A, resulting in an increase in pinene production to 8.1 ± 0.2 mg/L from 7.3 ± 0.2 mg/L (Figure 2B). Then, we integrated the *ttgB* from *P. putida* KT2440 under the control of the P37 promoter in *E. coli* YZ-3-A to obtain *E. coli* YZ-3-A-T. The modification further improved pinene production to 9.1 ± 0.2 mg/L (Figure 2B). These results indicate that overexpressing some efflux pumps (*E. coli acrAB* and *Pseudomonas putida* KT2440 *ttgB*), whcih improved pinene tolerance, also enhanced its production.

**Figure 3:**
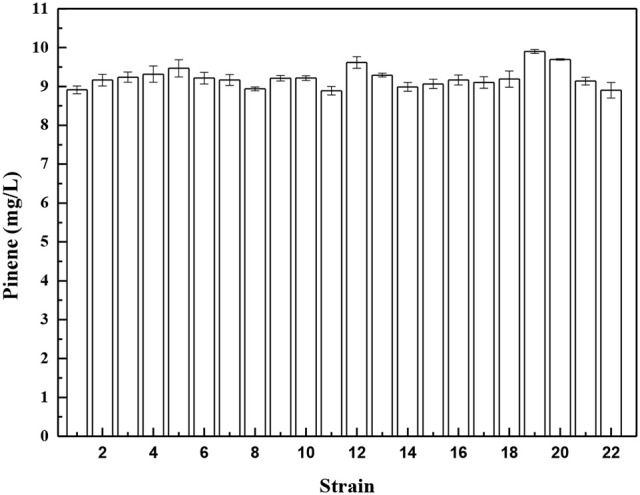
Pinene production of the selected mutants resistant to fosmidomycin harboring pMEVIGPS. *E. coli* BW25113 (P_T5_-dxs, pMEVIGPS) (strain No. 22) was set as the control strain. The data represent the means of three replicates and error bars represent standard deviations.

To further improve pinene production, we isolated a mutant resistant to an inhibitor of biosynthetic pathway after ARTP mutagenesis. Isolating a mutant resistant to an inhibitor of biosysnthetic pathway is a common strategy used for strain improvement. In *E. coli*, the important precursors IPP and DMAPP are produced by the 1-deoxy-D-xylulose-5-phosphate (DXP) pathway. Fosmidomycin is the DXP pathway inhibitor that inhibits 1-deoxy-D-xylulose-5-phosphate reductoisomerase (Dxr) and methylerythritol phosphate cytidyltransferase (IspD) of the DXP pathway (Zhang et al., 2011). Genes involved in the DXP pathway are essential for *E. coli* growth. The wild-type *E. coli* YZ-3-A-T can grow in the presence of 2% pinene (Supplementary Figure 2A), but does not grow in the presence of 35 μM fosmidomycin (Supplementary Figure 2B). After ARTP mutagenesis, cells grow well in the presence of 35 μM fosmidomycin (Supplementary Figure 2C). Overexpression of *dxr* or *ispD* in *E. coli* improved the fosmidomycin tolerance (Zhang et al., 2011). This indicates that the fosmidomycin resistant mutants may show higher level of Dxr and IspD. Screening the fosmidomycin resistant mutants will increase the probability to obtain a mutant with higher IPP flux. Thus, to increase the probability to obtain a mutant with higher IPP flux, we screened the fosmidomycin resistant mutants using the IPP/FPP sensor. *E. coli* YZ-3-A-T cells harboring pP_rstA_-GFP were treated with ARTP. After ARTP mutagenesis, the cells were transferred into the LB medium supplemented with 35 μM fosmidomycin and 2.0% pinene. A total of 720 clones were screened for analyzing fluorescence strength in deep-well microplate cultures (Supplementary Figure 3). Twenty-one strains with higher fluorescence strength were selected for further shake flask analysis. As shown in Figure 3, Strain No. 19, which was denoted as *E. coli* YZFP, produced the highest level of pinene, which reached 9.9 ± 0.1 mg/L. In fact, our quantitative real-time PCR analysis also demonstrates that the *dxs, dxr* and *ispD* of the DXP pathway in *E. coli* YZFP showed higher transcription level than the wild-type strain (Data not shown, will be published in another paper).

**Figure 4:**
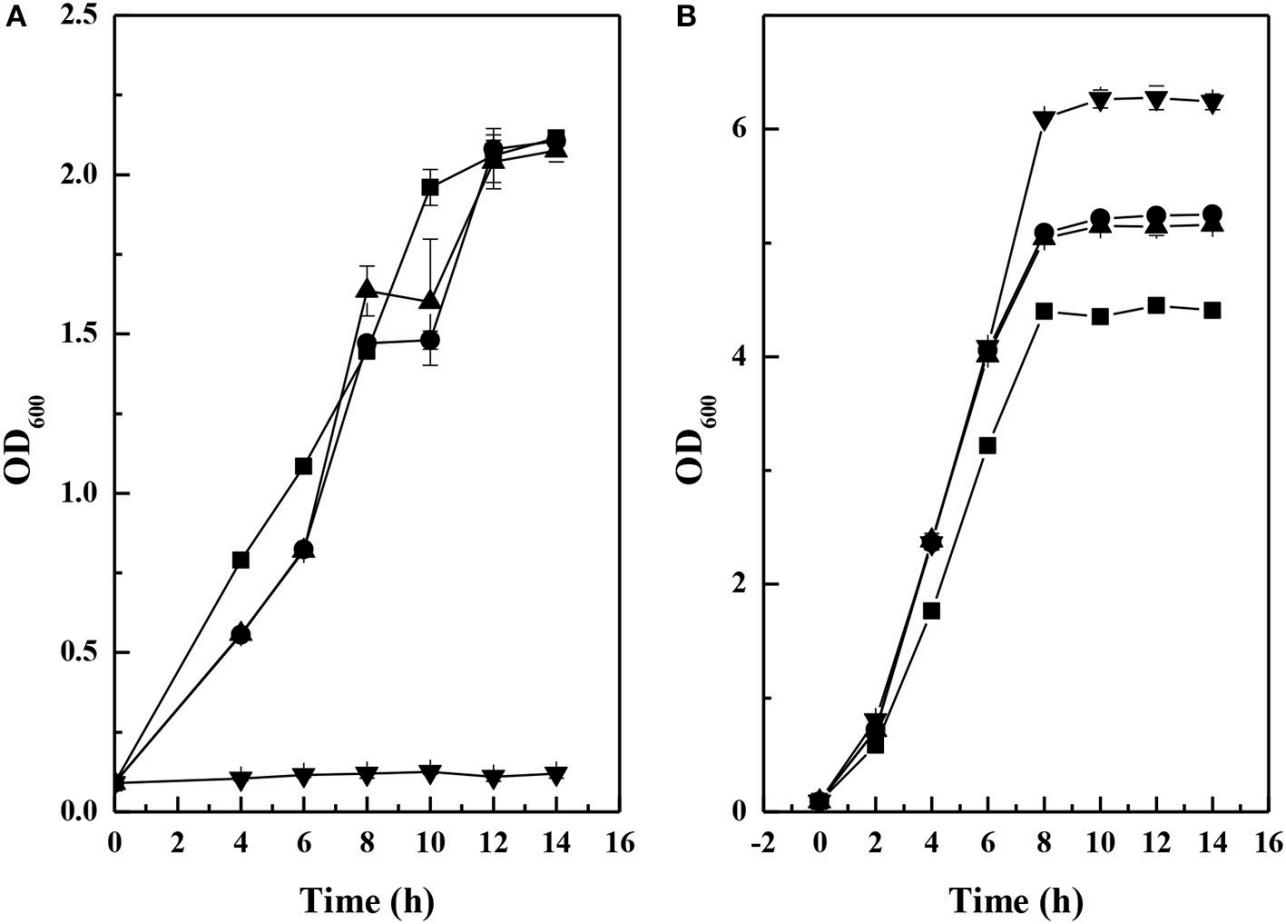
Growth of the selected tolerance strains in the presence of 2% pinene **(A)** and in the absence of pinene **(B)**. *E. coli* BW25113 (P_T5_-dxs) (▾), *E. coli* YZ-3 (▴), *E. coli* YZ-3-A-T (•), and *E. coli* YZFP (■). The data represent the means of three replicates and error bars represent standard deviations.

To characterize the pinene tolerance, the growth of the above strains were compared in different concentrations of pinene. Figure 4A shows the growths of these strains in the presence of 2% pinene. The starting strain did not grow well in the presence of 2% pinene. The above engineered strains did grow well in the presence of 2% pinene. The maximum cell densities of the three engineered strains were similar. The growth rate of *E. coli* YZFP was higher than that of the other engineered strains. These results indicate that the engineered strains have higher pinene tolerance than the starting strain. However, the maximum cell densities of the three engineered strains were lower than that of the starting strain in the absence of pinene (Figure 4B). The reason may be that the three engineered strains produced higher level of IPP than the starting strain. IPP is toxicity to *E. coli*. We also investigated the genetic stability of *E. coli* YZFP. The strain can also grow well in the presence of 2% pinene and the level of pinene production remained constant after 20 rounds of subculturing in absence of selective pressure (data not shown).

### Evolution engineering to improve pinene production

The lower expression level and/or lower enzymatic activity of GPPS and PS in *E. coli* may result in the lower yield of pinene production. Sarria et al. (2014) compared GPPSs and PSs from *A. grandis* and *P. taeda* and found that the combination of GPPS and PS from *A. grandis* was most suitable for pinene production. Thus, we first optimized the first 48 nucleotide sequences of *A. grandis GPPS* with the 6AA method to increase the expression level of the *A. grandis GPPS-PS* gene cluster in *E. coli*. The 6AA method substitutes all Arg, Asp, Gln, Glu, His, and Ile codons with the synonymous codon having the highest single-variable logistic regression slope (CGT, GAT, CAA, GAA, CAT, and ATT, respectively), while the other 14 amino acids were not changed from the wild-type gene sequence (Boë et al., 2016). The 6AA optimization increased pinene production from 5.6 ± 0.1 mg/L to 6.4 ± 0.3 mg/L (Table 2).

**Table 2:** Effect of evolution engineering on pinene production in *Escherichia coli* BW25113 (P_T5_-dxs, pMEVI).

**Gene cluster**	**Genetic modification**	**OD_600_**	**Pinene concentration (mg/L)**
*AgGPPS-AgPS*	Wild-type	12.30 ± 0.43	5.6 ± 0.1 (100.0%)
*AgGPPS_6*AA*_-AgPS*	The first 18 codons of *A. grandis* GPPS were optimized by using the 6AA method	12.22 ± 0.41	6.4 ± 0.3 (114.3%)
*AgGPPS_6*AA*_-AgPS^*epPCR*^*	The fusion *GPPS-PS* gene cluster variant from *A. grandis* after error-prone PCR	12.23 ± 0.39	10.4 ± 0.3 (185.7%)
*AgGPPS^*MUT*^-AgPS^*DNAshuffling*^*	The fusion *GPPS-PS* gene cluster variant from *A. grandis* after DNA shuffling	12.21 ± 0.45	12.4 ± 0.2 (221.4%)
*AgGPPS^*mut*^-Pt1^**Q*457*L**^*	The fusion gene cluster of the GPPS^D90G/L175P^ and Pt1^Q457L^	12.10 ± 0.38	15.2 ± 0.2 (271.4%)
*AgGPPS^*MUT*^-*TIGR*-Pt1^**Q*457*L**^*	The TIGR-mediated gene cluster of the GPPS and Pt1^Q457L^	12.11 ± 0.37	17.6 ± 0.2 (314.3%)

*Data represent the means of three replicates and standard deviations*.

**Figure 5:**
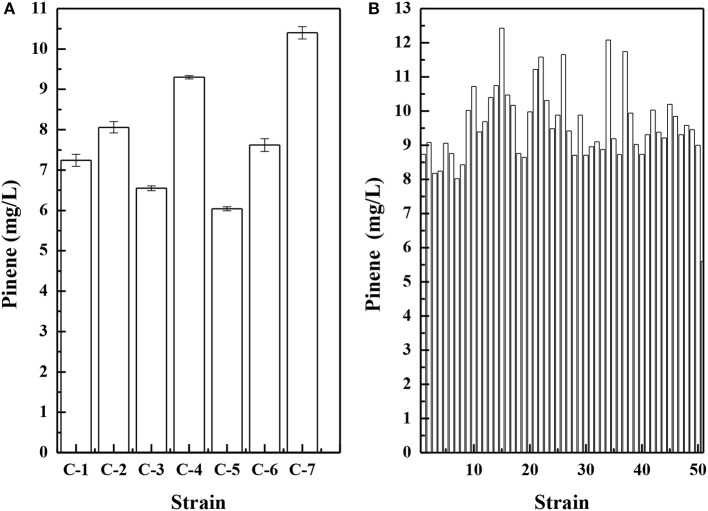
Pinene production by *E. coli* BW25113 (P_T5_-dxs, pMEVI) harboring mutant gene clusters from error-prone PCR **(A)** and by *E. coli* LYCOP harboring mutant gene clusters from DNA shuffling **(B)**. Pinene concentrations were measured using the GC-FID **(A)** and the concentrated sulfuric acid **(B)** methods. The data represent the means of three replicates and error bars represent standard deviations.

Because pinene shares the same 5-carbon precursors IPP and DMAPP with carotenoids, a lycopene-producing strain *E. coli* LYCOP (Chen et al., 2013) was used to screen the error-prone PCR mutant libraries of the GPPS-PS cluster from *A. grandis* after 6AA optimization. The higher the activity of the GPPS-PS cluster, the lower the intracellular precursor levels for lycopene biosynthesis, thereby reducing the pigmentation of the *E. coli*. Of approximately 1 500 colonies, 7 colonies with a whiter color were observed. The mutant plasmids were isolated from the 7 colonies and then were co-transferred with the MEV pathway plasmid pMEVI into *E. coli* BW25113 (P_T5_-dxs). The pinene productions of them were analyzed in a shake flask, and the results are presented in Figure 5A. The strains harboring the mutant gene cluster produced higher pinene by 7.8–85.7% than that with the wild-type gene cluster. To increase the gene cluster activity, the 7 mutant gene clusters were used for DNA shuffling.

Because the colonies of *E. coli* LYCOP harboring the above mutant gene cluster became a faint color, it is difficult to discriminate these colonies by using the above carotenoid-based method. A more sensitive and quantitative screening method is needed. It is known that monoterpene can hydrate readily in the presence of acid catalysts, such as H_2_SO_4_ (Robles-Dutenhefner et al., 2001). As a result, the initial reaction solutions turn yellow and then brown. After reaction with concentrated sulfuric acid in boiling water for 5 min, it was observed that the color of the reaction solution become darker as the pinene concentration increases and the absorbance at 450 nm is linearly related with pinene concentration (Supplementary Figure 4). Thus, the concentrated sulfuric acid method can quantitatively predict pinene concentrations.

After DNA shuffling, the mutant plasmids were transferred into *E. coli* LYCOP harboring pMEVI. Fifty colonies with a whiter color were used for assays of pinene production in a shake flask using the concentrated sulfuric acid method. The results are presented in Figure 5B. *E. coli* LYCOP harboring the mutant gene cluster produced higher pinene (6.5–10.1 mg/L) than those with the wild-type gene cluster. The mutant plasmid with the highest pinene production was isolated from strain No. 15 and then was co-transferred with pMEVI into *E. coli* BW25113 (P_T5_-dxs). *E. coli* BW25113 (P_T5_-dxs) harboring the mutant plasmid and pMEVI produced 12.4 ± 0.2 mg/L of pinene (Table 2). The mutant plasmid with the highest pinene production was then sequenced. The two amino acid mutants (D90G and L175P) were observed in the CDS of *GPPS* from *A. grandis*. No mutant in the CDS of *PS* from *A. grandis* was observed. It has been reported that (-)-α-pinene synthase (Pt1) from *P. taeda* has the lowest *K*_*m*_ for GPP among the known PSs (Phillips et al., 2003). Tashiro et al. engineered an *E. coli* with the highest yield of pinene so far using the pinene synthase mutant (Pt1^Q457L^) from *P. taeda* (Tashiro et al., 2016). Thus, replacing *A. grandis PS* in the mutant plasmid with *P. taeda* pinene synthase mutant gene (*Pt1*^*Q457L*^) yielded pQE30-GPPS^mut^-L-Pt1^Q457L^. *E. coli* BW25113 (P_T5_-dxs) harboring pQE30-GPPS^mut^-L-Pt1^Q457L^ produced a higher level of pinene (15.2 ± 0.2 mg/L) than those harboring pQE30-GPPS^mut^-L-AgPS, which achieved 12.4 ± 0.2 mg/L (Table 2).

**Figure 6:**
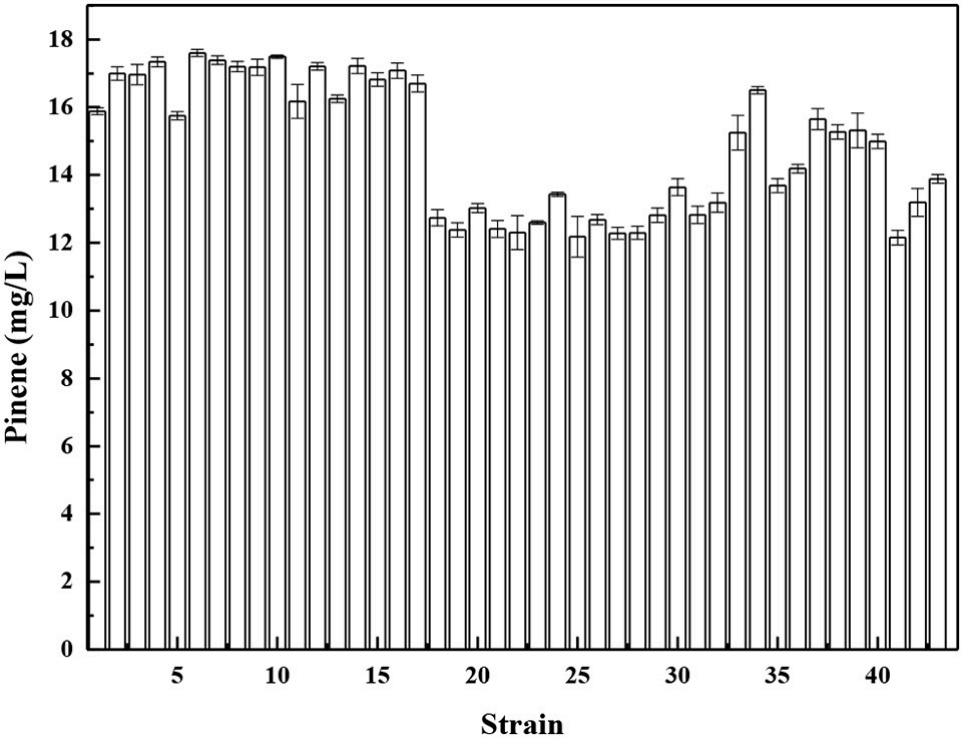
Pinene production by *E. coli* BW25113 (P_T5_-dxs, pMEVI) harboring the selected TIGR-mediated gene cluster. The data represent the means of three replicates and error bars represent standard deviations.

The unbalanced expression of multiple genes may overburden the cell and cause accumulation of toxic metabolic intermediates, resulting in reduced product titers. Pfleger et al. (2006) developed a combinatorial engineering approach for coordinating the expression of cascade enzymes. For this purpose, libraries of tunable intergenic regions (TIGRs) are generated that encode mRNAs with diverse secondary structures with RNase cleavage sites. The TIGR approach was applied to balance the gene expression of the MEV pathway using the TIGR approach, resulting in a 7-fold increase in mevalonate production. Moreover, our previous paper demonstrated that the TIGR approach was more efficient compared to protein fusion for coordinating expression (Li et al., 2015). Thus, we constructed a library of TIGRs to balance the expression of *A. grandis GPPS*^*D90G/L175P*^ and *P. taeda Pt1*^*Q457L*^. The library of TIGRS was inserted between *GPPS*^*D90G/L175P*^ and *P. taeda Pt1*^*Q457L*^ to yield a series of operons. The functional operons from the libraries were screened by using the concentrated sulfuric acid method. A total of 768 colonies were used for the assay of pinene production in deep-well microplate cultures using the concentrated sulfuric acid method (Supplementary Figure 5). Forty-three strains with higher OD_450_ were selected for further shake flask analysis. As shown in Figure 6, strain No. 6 produced the highest level of pinene (17.6 ± 0.2 mg/L). Thus, the TIGR-mediated plasmid was recovered from strain No. 6 and sequenced (Supplementary Table 2). We also retransformed the plasmid back to the host strain *E. coli* BW25113 (P_T5_-dxs) and checked the pinene production. The resulting strain produced the same level of pinene (17.9 ± 0.1 mg/L), indicating that the pinene production improvement is the result of TIGR-mediated optimization.

### Modular co-culture engineering to improve pinene production

**Figure 7:**
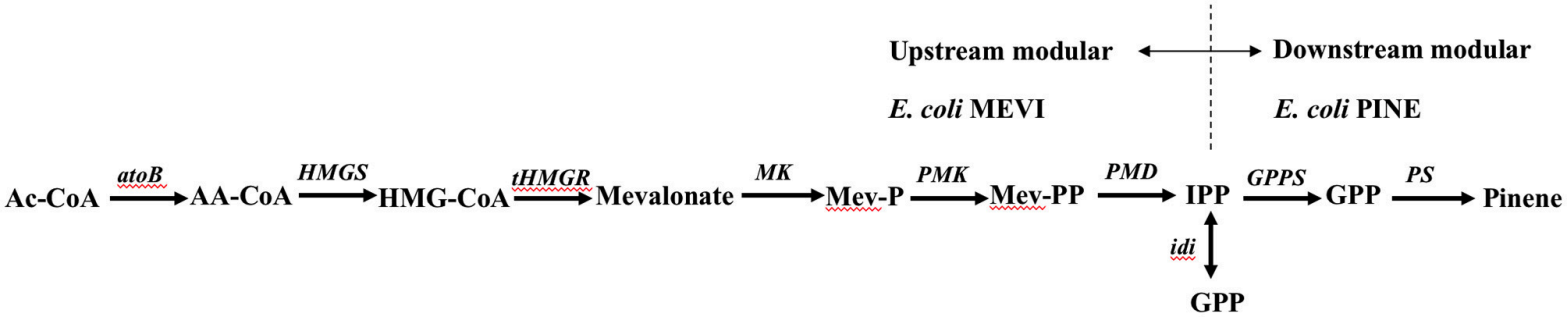
Strategy of modular co-culture engineering.

To take advantage of emerging co-culture engineering approaches to improve overall pinene biosynthesis in *E. coli*, the complete biosynthetic pathway was divided into the following two modules: the upstream module of the MEV pathway and the downstream module of the TIGR-mediated gene cluster of *A. grandis GPPS*^*Mut*^ and *P. taeda Pt1*^*MUT*^ (Figure 7). The two modules were integrated into the chromosome of the pinene tolerance strain *E. coli* YZFP and then then evolved to a higher gene copy number by triclosan induction, respectively.

**Figure 8:**
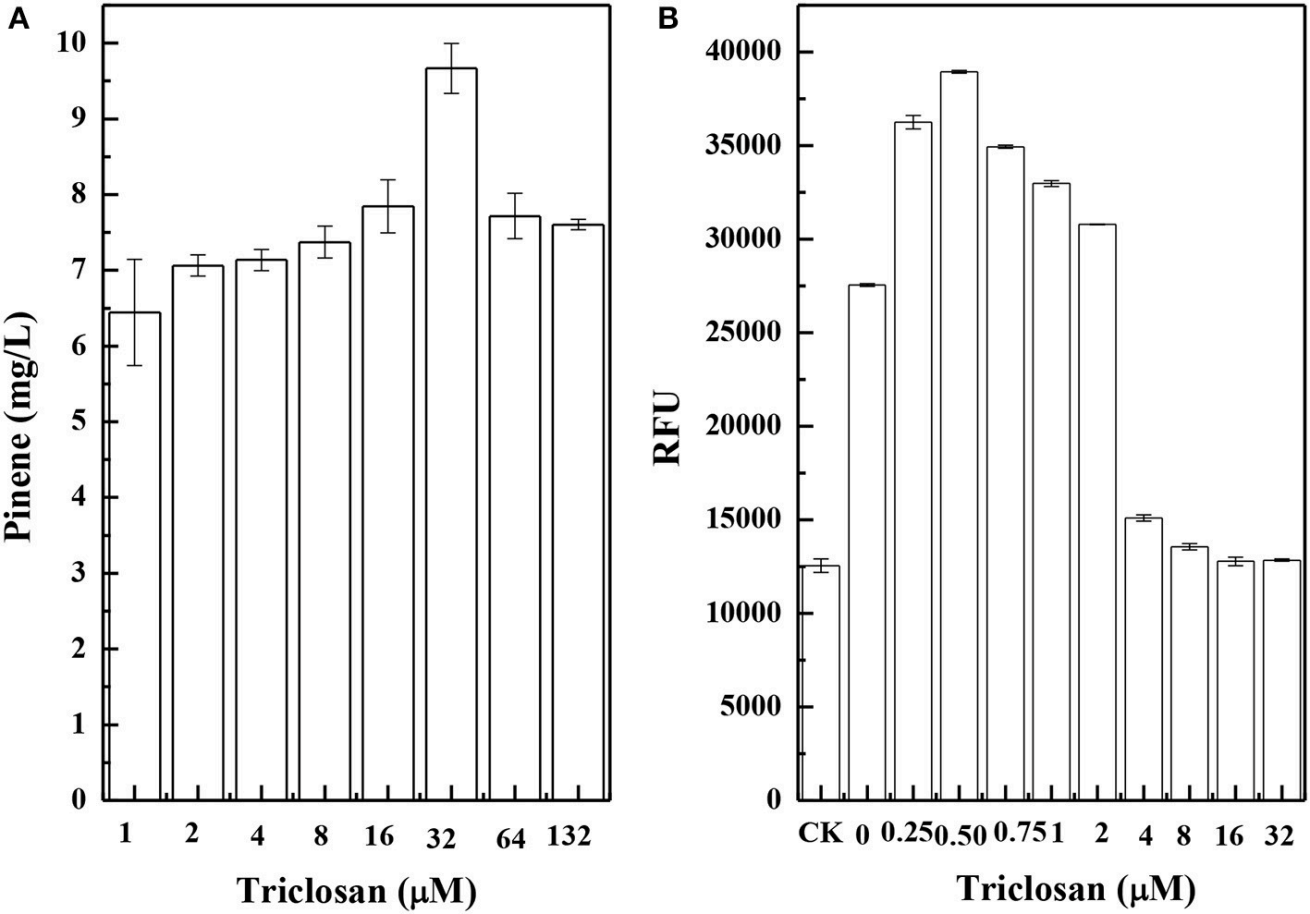
Pinene production of chemically induced chromosomal evolution (CIChE) strains of the GPPS-Pt1 cluster without the MEV pathway **(A)** and the MEV pathway **(B)** at different triclosan concentrations. The data represent the means of three replicates and error bars represent standard deviations.

Figure 8A shows the results of pinene production in CIChE strains of the TIGR-mediated gene cluster of *A. grandis GPPS*^*Mut*^ and *P. taeda Pt1*^*MUT*^ without the MEV pathway. The maximum pinene production was obtained by the CIChE strains resistant to 32 μM triclosan. Thus, the *recA* gene of the CIChE strain resistant to 32 μM triclosan was deleted to obtain *E. coli* PINE. We determined the *GPPS-Pt1* gene copy number in *E. coli* PINE. The copy number reached approximately 60 in the CIChE strain, which is the equivalent copy number of a high copy plasmid. Figure 8B shows the results of IPP/FPP concentration of the CIChE strains of the MEV pathway measured by the IPP/FPP sensor (pP_rstA_-GFP). As shown in Figure 8B, the maximum IPP/FPP production was obtained by the CIChE strains resistant to 0.5 μM triclosan. Thus, the *recA* gene of the CIChE strain resistant to 0.5 μM triclosan was deleted to obtain *E. coli* MEVI. We also determined the MEV pathway gene copy number in *E. coli* MEVI. The copy number reached approximately 4 in *E. coli* MEVI.

**Figure 9:**
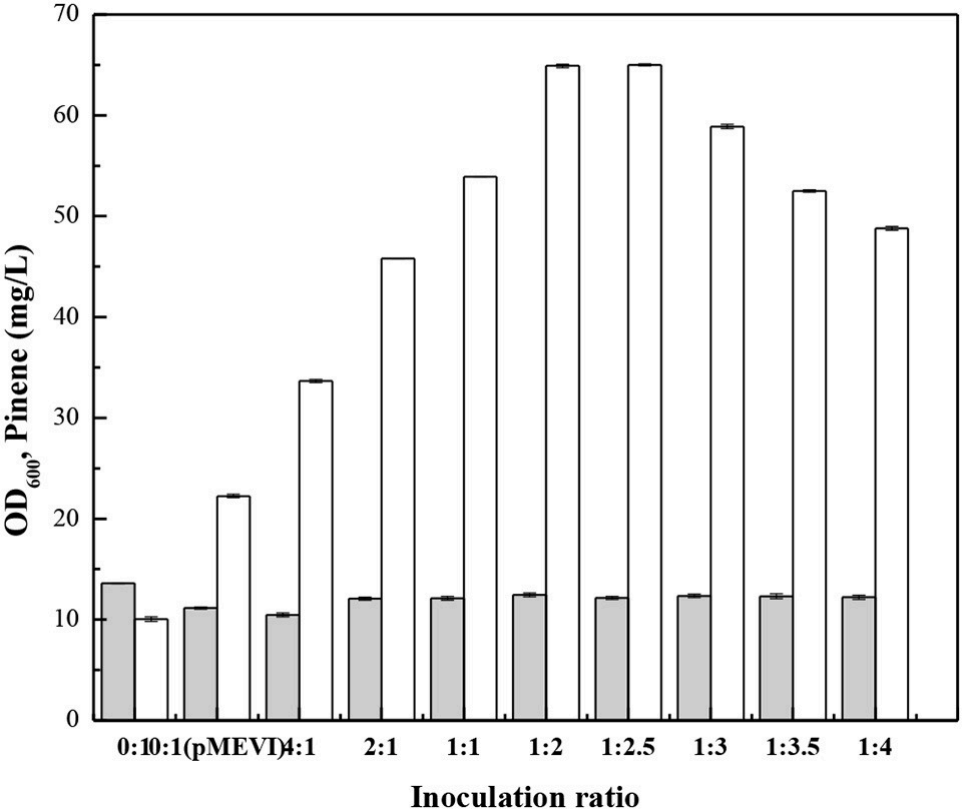
Effect of the inoculation ratio of *E. coli* PINE and MEVI on pinene production in the co-culture system. OD_600_ (Gray bars), Pinene concentration (White bars). 0:1, only *E. coli* PINE; 0:1 (pMEVI), only *E. coli* PINE (pMEVI); others, the *E. coli* MEVI: PINE co-culture system with different inoculation ratio. The data represent the means of three replicates and error bars represent standard deviations.

Zhou et al. (2015) demonstrate that the modular co-culture engineering can be applicable to isoprenoids because their scaffold moleculars can generally permeate membranes. To demonstrate IPP can also cross cell membranes, we cultured *E. coli* (pP_rstA_-GFP) with the cell-free culture broth of *E. coli* MEVI and measured fluorescence strength. After addition of the cell-free culture broth of *E. coli* MEVI, *E. coli* (pP_rstA_-GFP) showed higher fluorescence strength (Supplementary Figure 6). Moreover, the *E. coli* MEVI: PINE co-culture produced higher level of pinene than *E. coli* PINE (Figure 9). These results indicate that IPP produced by *E. coli* MEVI diffused into *E. coli* PINE and was subsequently converted into pinene. We then optimized the *E. coli* MEVI: PINE co-culture system to further improve pinene production. To this end, different inoculation ratios between *E. coli* MEVI and PINE were investigated. As shown in Figure 9, the highest pinene production of 64.9 ± 0.9 mg/L was achieved when *E. coli* MEVI and PINE were inoculated at a ratio of 1:2. Compared with the mono-culture strategy using *E. coli* PINE harboring pMEVI, the pinene production was increased by 1.9-fold (from 22.3 ± 0.2 mg/L to 64.9 ± 0.9 mg/L). To test if all of IPP produced by *E. coli* MEVI were converted into pinene by *E. coli* PINE, we measured the IPP concentration in the broth of the co-culture and the *E. coli* MEVI after 28 h using the IPP sensor plasmid. The results showed that about 57.8% of IPP were converted by *E. coli* PINE (Supplementary Table 3). Thus, we introduced pQE-GPPS^MUT^-TIGR-Pt1^Q457L^ to overexpress the pinene biosynthetic pathway and checked the pinene production. The co-culture system after introducing the pinene biosynthetic pathway into *E. coli* MEVI produced higher level of pinene (60.2 ± 0.2 mg/L) than the control strain (52.1 ± 0.1 mg/L) harboring the empty plasmid (Supplementary Table 4). The result also demonstrates that not all of IPP can be converted in the co-culture system.

**Figure 10:**
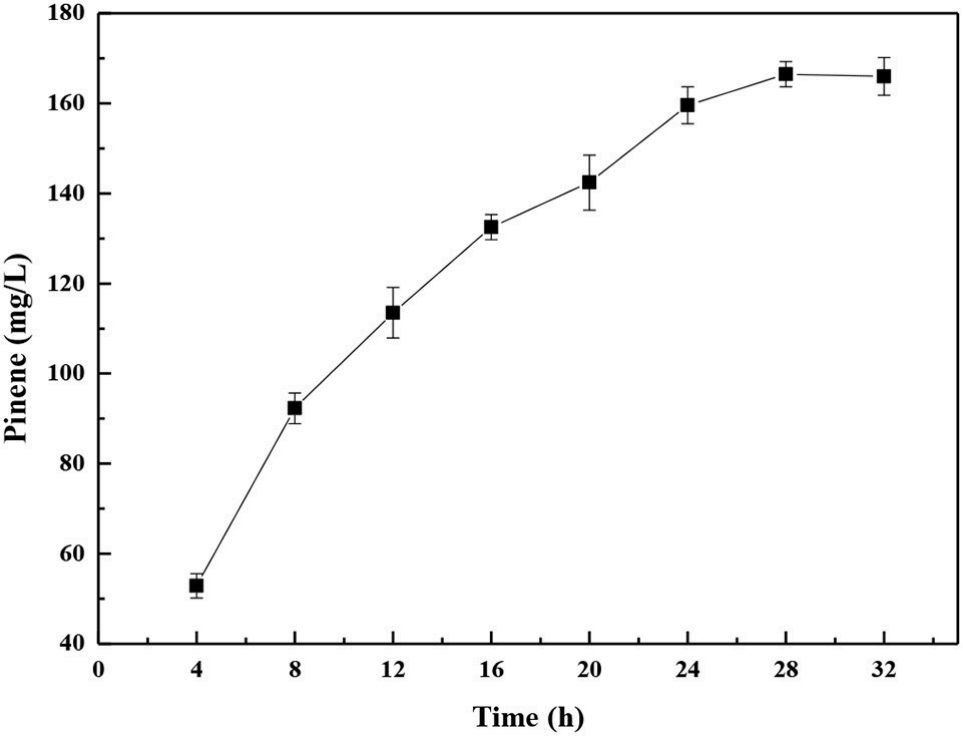
Time course of pinene production by the modular co-culture system of the whole-cell biocatalyst. The data represent the means of three replicates and error bars represent standard deviations.

Biotechnological approaches for chemicals production can be broadly classified into fermentation and biocatalysis. In biocatalysis, cell growth and production phase are separated. In comparison to the fermentation bioprocess, whole-cell biocatalysis is an attractive method due to its great efficiency and relative simplification of downstream processing (Lin and Tao, 2017). The whole-cell biocatalysis processes comprise the following two stages: growth and conversion of the substrates. After the cells are cultured, they are harvested and washes with a buffer solution and suspended in the buffer for biocatalysis. Thus, the *E. coli-E. coli* modular co-culture system of whole-cell biocatalysis was used to further enhance pinene production. As shown in Figure 10, the highest pinene production of 166.5 ± 0.3 mg/L was achieved by the whole-cell biocatalyst after 28 h. The pinene titer obtained by the whole-cell biocatalysis was 2.6-fold higher than that produced by the fermentation process.

## Discussion

It has been reported that *E. coli* growth is inhibited by 0.5% pinene (Dunlop et al., 2011). We first improved pinene tolerance from 0.5 to 2.0% and pinene production by adaptive laboratory evolution after ARTP mutagenesis. In fact, improvements in tolerance are not sufficient to guarantee an increase production. Our results also demonstrate this point. Overexpreesion of *A. borkumensis acrBDFa* or *P. putida* KT2440 *mexF* that improved pinene tolerance did not improved pinene production (Figure 2A). To obtain a mutant with higher level of pinene production, we used the IPP/FPP sensor pP_rstA_-GFP to screen the mutants tolerant to 2% pinene. Tolerance engineering has also successfully been used to improve the production of limonene (Dunlop et al., 2011), amorphadiene (Zhang et al., 2016), olefin (Mingardon et al., 2015), n-octane (Foo and Leong 2013). Although the level of pinene production reported in literatures did not inhibit growth, higher tolerance is beneficial to pinene production. Thus, the 2% pinene tolerant strain *E. coli* was used the parent strain in this study. To further improve pinene production, we then expressed the efflux pumps in the pinene tolerant strain *E. coli* and subsequently selected a mutant resistant to fosmidomycin after ARTP mutagenesis. The pinene tolerant strain *E. coli* YZFP with higher level of pinene production was obtained through a two-step screening process. There is no directed evidence to prove the improved pinene production is the result of improved pinene tolerance.

Our study demonstrates that the overexpression of some efflux pumps improved pinene tolerance and production. Many groups also reported that overexpression of efflux pumps enhanced biofuel tolerance. Dunlop et al. (2011) reported that the overexpression of efflux pumps, such as *A. borkumensis* AcrBDFa, *P. putida* KT2440 MexF, *P. putida* KT2440 TtgB or *E. coli* AcrB, enhanced pinene tolerance. However, they did not investigate the effects of the pumps on pinene production. Our results demonstrate that overexpression of *E. coli* AcrAB and *P. putida* KT2440 TtgB enhanced pinene production (Figure 2). Overexpression of *A. borkumensis* AcrBDFa or *P. putida* KT2440 MexF did not improved pinene production (Figure 2A). However, Dunlop et al. (2011) reported that overexpression of *A. borkumensis* AcrBDFa enhanced limonene tolerance and yield. Overexpression of *tolC* together with ABC family transporters (*macAB*) or MFS family transporters (*emrAB* or *emrKY*) was found to improve amorphadiene titer by more than 3-fold (Zhang et al., 2016). Overexpression of the native and evolved *acrB* improved olefin tolerance and production (Mingardon et al., 2015). Evolved AcrB variants with improved tolerance to pinene and n-octane have also been reported by Foo and Leong (2013). Taken together with these previous studies, our results show that a combination of the adaptive laboratory evolution with overexpression of some efflux pumps can improve pinene tolerance and production.

In this study, we reported a high-throughput screening method, which is known as the concentrated sulfuric acid method, for recombinant *E. coli* that overproduce pinene. We successfully applied the concentrated sulfuric acid method to screen the DNA shuffling library of the GPPS-PS gene cluster and the library of the TIGR-mediated *GPPS-Pt1* gene cluster. Because limonene has the same properties as pinene, the concentrated sulfuric acid method can also be used to screen mutants for limonene production. Although the carotenoid-based method has been successfully used to screen isoprene synthase variants (Emmerstorfer-Augustin et al., 2016), the carotenoid-based method has a limitation when the colony has a faint color.

GPPS and PS have been identified as a major limiting factor in pinene production (Yang et al., 2013; Sarria et al., 2014; Tashiro et al., 2016). After directed evolution of the *A. grandis GPPS-PS* gene cluster using error-prone PCR and DNA shuffling, pinene production was increased by 1.2-fold (Table 2). Two amino acid mutants were observed in the CDS of *A. grandis GPPS*. However, no mutant was observed in the CDS of *A. grandis PS*. Tashiro et al. evolved *P. taeda Pt1* and constructed a recombinant *E. coli* with the highest pinene yield reported in literatures using the evolved variant (Tashiro et al., 2016). Using the *A. grandis GPPS*^*Mut*^-*P. taeda Pt1*^*MUT*^ gene cluster resulted in an increase in pinene production by 22.6% compared to using the *A. grandis* GPPS^Mut^-PS gene cluster (Table 2).

GPPS and PS are inhibited by their substrate (GPP) or product (pinene) (Sarria et al., 2014). To overcome GPPS inhibition by GPP, GPPS was fused to PS, resulting in improved pinene production (Sarria et al., 2014; Tashiro et al., 2016). Our previous paper demonstrated that the TIGR approach was more efficient compared to protein fusion for coordinating expression (Li et al., 2015). This study shows that using the TIGR-mediated gene cluster led to an increase in pinene production by 15.8% compared with the fused gene cluster (Table 2).

In the present study, an *E. coli-E. coli* co-culture system was engineered to modularize the MEV and heterologous biosynthetic pathway. The MEV pathway and heterologous biosynthetic pathway (the *A. grandis GPPS*^*Mut*^-*P. taeda Pt1*^*MUT*^ gene cluster) was engineered in the pinene tolerance strain *E. coli* YZFP, respectively. The best co-culture system was found to improve pinene production by 1.9-fold compared to the mono-culture system. The modular co-culture can distribute the metabolic burden and allow for modular optimization by simply changing the strain-to-strain ratio. The *E. coli-E. coli* modular co-culture system has been successfully used to improve 3-amino-benzoic acid (Zhang and Stephanopoulos, 2016), flavonoid (Jones et al., 2016), muconic acid (Zhang et al., 2015), and perillyl acetate (Willrodt et al., 2015), etc. In fact, the critical issue for modular co-culture engineering is the mass transfer of the pathway intermediate (IPP). It has been demonstrated that isoprenoids scaffold molecules can cross cell membranes (Zhou et al., 2015). Our results also demonstrate that IPP can cross cell membranes and secreted to the extracellular medium (Supplementary Figure 6 and Figure 9). Moreover, our results showed that the pinene tolerance strain *E. coli* YZFP (pP_rstA_-GFP) had higher fluorescence strength than the parent strain harboring pP_rstA_-GFP after addition the cell-free broth of *E. coli* MEVI (Supplementary Figure 6), indicating that *E. coli* YZFP shows greater membrane permeability than the parent strain. Our results demonstrate that there were still some IPP not to be converted into pinene by *E. coli* PINE (Supplementary Tables 3, 4). Moreover, Overexpression of the pinene biosynthetic pathway in *E. coli* MEVI enhanced pinene production in the *E. coli* MEVI-*E. coli* PINE co-culture system (Supplementary Table 4). However, overexpression of the pinene biosynthetic pathway in *E. coli* PINE did not enhance pinene production (Supplementary Table 4). Increasing the inoculation ratio of *E. coli* PINE and *E. coli* MEVI from 2:1 to 2.5:1 or 3:1 did not enhanced pinene production (Figure 9). These results indicate that the IPP transportation may be a key factor for further improving pinene production. Transporter engineering strategies have successfully been used to enhance the secretion of the pathway intermediates, improving production (Boyarskiy and Tullman-Ercek, 2015; Kell et al., 2015; Zhang et al., 2015). Thus, appropriate metabolite transporters engineering strategies may be used to further improve pinene production of the *E. coli-E. coli* co-culture system.

This study also demonstrated that whole-cell biocatalysis further improved pinene production by 1.6-fold compared to the fermentation process. The whole-cell biocatalysis has also successfully been used in many biotechnological production (Tao et al., 2011; Lin et al., 2015; Kogure et al., 2016; Chen et al., 2017; Lin and Tao, 2017). Kogure et al. (2016) also reported that the significantly higher shikimate productivity (141.3 g/L) was achieved by the whole-cell biocatalysis compared to that (78.8 g/L) achieved by the fed-batch fermentation accompanying cell growth. The pinene production improvement may be resulted from higher cell density (OD_600_ of 30) and the growth-arrested cells used in the whole-cell biocatalysis.

## Conclusions

Pinene tolerance and production were first improved via adaptive laboratory evolution and efflux pump overexpression. Through error-prone PCR and DNA shuffling, a GPPS variant was screened, which outperformed the wild-type enzyme. To balance the expression of multiple genes, a TIGR was inserted between *A. grandis* GPPS^D90G/L175P^ and *P. taeda* Pt1^Q457L^. To construct an *E. coli-E. coli* co-culture system to modularize the MEV and heterologous biosynthetic pathway, the MEV pathway and heterologous biosynthetic pathway (the *A. grandis GPPS*^*Mut*^-*P. taeda Pt1*^*MUT*^ gene cluster) was integrated into the chromosome of the pinene tolerance strain *E. coli* YZFP and then evolved to a higher gene copy number by CIChE, respectively. The *E. coli-E. coli* modular co-culture system of whole-cell biocatalysis resulted in the highest pinene production of 166.5 mg/L. Our results demonstrate that the *E. coli-E. coli* modular co-culture system of the whole-cell biocatalysis is a promising approach for the production of pinene.

## Author contributions

F-XN performed all of the experimental works. XH and Y-QW performed the pinene assay. J-ZL designed the study and wrote the manuscript. All the authors read and approved the final manuscript.

### Conflict of interest statement

The authors declare that the research was conducted in the absence of any commercial or financial relationships that could be construed as a potential conflict of interest.
