# Supplementary material for: Enhancing Production of Pinene in Escherichia coli by Using a Combination of Tolerance, Evolution, and Modular Co-culture Engineering
Source: Front Microbiol. 2018 Jul 31;9:1623. doi: 10.3389/fmicb.2018.01623 (PMC6079208; doi:10.3389/fmicb.2018.01623)
Supplement: Supplementary file 1 [file Table_1.DOCX]

Suppl. Table 1 Primers used in this study

| Name | Sequence | Purpose |
| --- | --- | --- |
| EcoRI-GPPS | CCGGAATTCTTTCGGAATTAAGGAGGTAAT (BamHI) | Error-prone PCR |
| HindIII-PS | CCCAAGCTTTTACAGCGGAACGGACTCC (HindIII) |  |
| acrAF | CGCGGATCCATGAACAAAAACAGAGGGTTT (BamHI) | PCR for *E. coli* *acrAB* |
| acrBR | CCCAAGCTT TCAATGATGATCGACAGTA (HindIII) |  |
| mexFF | CGCGGATCCATGAACTTCTCGAAATTCTTC (BamHI) |  |
| mexFR | CCCAAGCTTTGCGTGGTTCTCCAGCACTTG (HindIII) |  |
| acrBDFaF | CGCGGATCCATGGCCAGATTTTTTATTG (BamHI) | PCR for the *acrBDFa* |
| acrBDFaR | CCCAAGCTTTTATTCTTTGCCTTTCTTCT (HindIII) |  |
| ttgBF | CGCGGATCCATGTCGAAGTTCTTTATCGAT (BamHI) | PCR for the *ttgB* |
| ttgBR | CCCAAGCTTTCATTGCCCAGCCTCATAAC (HindIII) |  |
| AcrA-R | GCTAACTTGAGGACGAAC | PCR for homologous arm for replacement of *acrAB* promoter |
| TH-PAB1 | GTTTCTTGCGCTTCTTGTTTGGTTTTTCGTGCCATAGGCCCTTTCGTCTTCAC |  |
| TH-PAB1 | AAAGACGTAGAGCCACATCGAGGATGTGTTGGCGCGTTTCTTGCGCTTCTTGT |  |
| AB-N20 | TTTATATTATCGTCGTGCTA | N20 for *acrAB* |
| 5XhoI-SS9 | CCGCTCGAGGCTTGGTTGAGAATACGCCG (XhoI) | PCR for homologous arm for the integration of the *ttgB* at the SS9 site |
| 5BglII-SS9 | GGAAGATCTGTTTGACATATCAACTGCGCCAG (BglII) |  |
| 3KpnI-SS9 | CGGGGTACCTTATTATATCGCGTTGATTA (KpnI) |  |
| 3ApaI-SS9 | GACGGGCCCGCCTACGATTACGCATGGCT (ApaI) |  |
| SS9-N20 | TCTGGCGCAGTTGATATGTA | N20 for SS9 |
| QPt1F | CTTATTTGCCTCGTTTGGAAGC | qPCR for *Pt1* |
| QPt1R | CCACCAACGAACCAAATATTCC |  |
| QHF | GCTACAGATACAGCACGTTA | qPCR for *HMGS* |
| QHR | CAGTGTAAGCAACAGCATCT |  |
| GPPSR | CCGGAGCTCTTAATTCTGACGAAATGCC (SacI) | PCR for pQE-GPPS^MUT^-Pt1 ^Q457L^ constuction |
| Pt1F | AGTGTCGACTTTCGGAATTAAGGAGGTAAT (SalI) |  |
| Pt1R | CCCAAGCTT TTACAAAGTAACAGGATC (HindIII) |  |
| Primers for construction of TIGRs library | | |
| TIGR-F | CCGGAGCTCGCCTAGCAAGATCTCCTGATC | PCR for TIGRs library(SacI) |
| TIGR-R | ACGCGTCGACGGATACAGTATCTGCGGTACC | PCR for TIGRs library (SalI) |
| A1 | 5’-GCCTAGCAAGATCTCCTGATCCCGGTGCACCCGGACATCTGCATAGTCTG-3’ | |
| A2 | 5’-GCCTAGCAAGATCTCCTGATCCACCCGGACATCTGCATAGTCTG-3’ | |
| A3 | 5’-GCCTAGCAAGATCTCCTGATCCCGGTGCGCGACCACCCGGACATCTGCATAGTCTG-3’ | |
| A4 | 5’-GCCTAGCAAGATCTCCTGATCCACATAAGGCCGGACATCTGCATAGTCTG-3’ | |
| A5  A6  A7  A8  A9  A10  A11  B1  B2  B3  B4  B5  B6  B7  B8  B9  B10  C1  C2  C3  C4  C5  C6  C7  C8  C9  C10  D1  D2  D3  D4  D5  D6  D7  D8  D9 | 5’-GCCTAGCAAGATCTCCTGATCCACCTTTGATGGCTAGAAAAATTAAGCTGCGGACATCTGCATAGTCTG-3’  5’-GCCTAGCAAGATCTCCTGATCCNNNNNCACCCGGACATCTGCATAGTCTG-3’  5’-GCCTAGCAAGATCTCCTGATCCACCTTGAGAGTACTTAATGTAAGCCCTCTCTCAGACATCTGCATAGTCTG-3’  5’-GCCTAGCAAGATCTCCTGATCAGAGGGACAANNNNAAGGTCATTCAGACATCTGCATAGTCTG-3’  5’-GCCTAGCAAGATCTCCTGATCAGAGGGACAANNNNNAAGGTCATTGCAGCTCAGACATCTGCATAGTCTG-3’  5’-GCCTAGCAAGATCTCCTGATCAGAGGGACAANNNNAAGGTCATTGCAGCTCAGACATCTGCATAGTCTG-3’  5’-GCCTAGCAAGATCTCCTGATCAGAGGGACAANNNNNAAGGTCATTGCAGCTCAGACATCTGCATAGTCTG-3’  5’-AAATACTGTAAATTCAAGGCAAGTGTACCTGATCCCGGTGCACCCAGACTATGCAGATGT-3’  5’-AAATACTGTAAATTCAAGGCAAGTGTAAGTGTACCTGATCCACCCAGACTATGCAGATGT-3’  5’-AAATACTGTAAATTCAAGGCACCTGATCCCGGTGCGCGACCACCCAGACTATGCAGATGT-3’  5’-AAATACTGTAAATTCAAGGCCCTGATCCGCCAGTCCTCAGACTGGCCCAGACTATGCAGATGT-3’  5’-AAATACTGTAAATTCAAGGCACCTTTTATGGACGACTAGAAAATTAAACTGCAGACTATGCAGATGT-3’  5’-AAATACTGTAAATTCAAGGCACCTTGAGAGTACTTAAGGTAAGCCCTCTCTCAGACTATGCAGATGT-3’  5’-AAATACTGTAAATTCAAGGCAGAAGGTCAANNNNNAAGGCCACTCAGACTATGCAGATGT-3’  5’-AAATACTGTAAATTCAAGGCACCTTGAGAGTACTTAAGGTAAGCCCTCTCTCAGACTATGCAGATGT-3’  5’-AAATACTGTAAATTCAAGGCAGAAGGTCAANNNNNAAGGCCACTGCAGCTCAGACTATGCAGATGT-3’  5’-AAATACTGTAAATTCAAGGCAGAAGGTCAANNNNAAGGCCACTGCAGCTCAGACTATGCAGATGT-3’  5’-GCCTTGAATTTACAGTATTTATTTGTATTGATCTCCTTATCCGCTCAAGA-3’  5’-GCCTTGAATTTACAGTATTTGATCTCAATGCTCTATCAATGTAGGAAGATACCTTATCCGCTCAAGA-3’  5’-GCCTTGAATTTACAGTATTTCAGTTACCGCTCTATCCTTATCCTTATCCGCTCAAGA-3’  5’-GCCTTGAATTTACAGTATTTAATTTACCTTTGATTTCCGGATCCTTATCCGCTCAAGA-3’  5’-GCCTTGAATTTACAGTATTTACAAGTTTTGATCGAGGGACAGTAGTCCTTATCCGCTCAAGA-3’  5’-GCCTTGAATTTACAGTATTTAGCGTTCCGAGTGCATGCCTTATCCGCTCAAGA-3’  5’-GCCTTGAATTTACAGTATTTAGCGTTCCGAGTGCATGCTCGCCCTTATCCGCTCAAGA-3’  5’-GCCTTGAATTTACAGTATTTACCGAGTGCATGCCTTATCCGCTCAAGA-3’  5’-GCCTTGAATTTACAGTATTTAATGAACTAGCGTTCCGAGTGCATG CCTTATCCGCTCAAGA-3’  5’-GCCTTGAATTTACAGTATTTTAGTGGCCTTNNNNNATACTATTCGGTCA CCTTATCCGCTCAAGA-3’  5’-GGATACAGTATCTGCGGTACCGATCTCCAATATCCGCTCTATTCTTGAGCGGATAAGG-3’  5’-GGATACAGTATCTGCGGTACCGATCTCCAAGATCCGATTATATTCTTGAGCGGATAAGG-3’  5’-GGATACAGTATCTGCGGTACCACCTTTTCTTGATTTCCGAATCCTATCCACTCAATTTTCTTGAGCGGATAAGG-3’  5’-GGATACAGTATCTGCGGTACCGATATCCTAGCGGATCCTATTAACTCTCCGCTTCTTGAGCGGATAAGG-3’  5’-GGATACAGTATCTGCGGTACCGATCGGGCCGGTATCCGGTTATTCTTGAGCGGATAAGG-3’  5’-GGATACAGTATCTGCGGTACCCTAGATGCGTTCCGAGTGCATGCTCGCTTCTTGAGCGGATAAGG-3’  5’-GGATACAGTATCTGCGGTACCCTAGATGCTTCCGAGTGCATGCTCGCTTCTTGAGCGGATAAGG-3’  5’-GGATACAGTATCTGCGGTACCCTAGTCCGAGTGCATGTTCTTGAGCGGATAAGG-3’  5’-GGATACAGTATCTGCGGTACCCTAGATGCGTTCCGAGTGCATGTTCTTGAGCGGATAAGG-3’ | |
