# Supplementary material for: Enhancing Production of Pinene in Escherichia coli by Using a Combination of Tolerance, Evolution, and Modular Co-culture Engineering
Source: Front Microbiol. 2018 Jul 31;9:1623. doi: 10.3389/fmicb.2018.01623 (PMC6079208; doi:10.3389/fmicb.2018.01623)
Supplement: Supplementary file 2 [file Table_2.DOCX]

Suppl. Table 2 The sequence of the selected TIGR

gagctcgcctagcaagatctcctgatccacccggacatctgcatagtctgggtgcaccgggatcaggtacacttgccttgaatttacagtatttagcgttccgagtgcatgccttatccgctcaagagcggagagttaataggatccgctaggatatcggtaccgcagatactgtatccgtcga
