# Supplementary material for: Enhancing Production of Pinene in Escherichia coli by Using a Combination of Tolerance, Evolution, and Modular Co-culture Engineering
Source: Front Microbiol. 2018 Jul 31;9:1623. doi: 10.3389/fmicb.2018.01623 (PMC6079208; doi:10.3389/fmicb.2018.01623)
Supplement: Supplementary file 3 [file Table_3.DOCX]

Suppl. Table 3 IPP concentrations measured by using the IPP sensor plasmid

| Culture broth | Relative fluorescence strength | Convert rate of IPP (%) |
| --- | --- | --- |
| *E. coli* MEVI | 9672.6±47.4 |  |
| *E. coli* MEVI: PINE co-culture | 4102.2±231.4 | 57.8 |

*E. coli* harboring pP_rstA_-GFP was incubated at 30°C and 130 rpm until an OD_600_ of 4.0 was reached. Then the cell-free culture broth of *E. coli* MEVI or *E. coli* MEVI: PINE co-culture cultured after 28 h was added with the ratio of 1:1. The cultures were incubated for additional 12 h and then the fluorescence strengths were assayed.
