# Supplementary material for: Enhancing Production of Pinene in Escherichia coli by Using a Combination of Tolerance, Evolution, and Modular Co-culture Engineering
Source: Front Microbiol. 2018 Jul 31;9:1623. doi: 10.3389/fmicb.2018.01623 (PMC6079208; doi:10.3389/fmicb.2018.01623)
Supplement: Supplementary file 4 [file Table_4.DOCX]

Suppl. Table 4 Effect of overexpression of the pinene biosynthetic pathway on pinene production in the co-culture system of fermentation

| Strain | OD | Pinene (mg/L) |
| --- | --- | --- |
| *E. coli* MEVI(pQE30): *E. coli* PINE(pQEA) | 12.43±0.25 | 52.1±0.1 |
| *E. coli* MEVI (pQE-GPPS^MUT^-TIGR-Pt1^Q457L^): *E. coli* PINE (pQEA) | 12.15±0.25 | 60.2±0.2 |
| *E. coli* MEVI (pQEA): *E. coli* PINE (pQE-GPPS^MUT^-TIGR-Pt1^Q457L^) | 12.22±0.34 | 53.9±0.1 |

pQEA is the pQE30 derivative with the p15 *ori.*
