# Supplementary material for: Enhancing Production of Pinene in Escherichia coli by Using a Combination of Tolerance, Evolution, and Modular Co-culture Engineering
Source: Front Microbiol. 2018 Jul 31;9:1623. doi: 10.3389/fmicb.2018.01623 (PMC6079208; doi:10.3389/fmicb.2018.01623)
Supplement: Supplementary file 5 [file Image_1.pdf]

Enhancing production of pinene in *Escherichia coli* by using a combination of tolerance, evolution and modular co-culture engineering

*Fu-Xing Niu, Xin He, Ya-Qin Wu, Jian-Zhong Liu**

Institute of Synthetic Biology, Biomedical Center, Guangdong Province Key Laboratory of Improved Variety Reproduction in Aquatic Economic Animals and South China Sea Bio-Resource Exploitation and Utilization Collaborative Innovation Center, School of Life Sciences, Sun Yat-sen University, Guangzhou 510275, China

*Corresponding author: Institute of Synthetic Biology, School of Life Science, Sun Yat-Sen University, Guangzhou 510275, P.R. China. Phone: +86-20-84110115. Fax: +86-20-84036461. *E-mail address*: lssljz@mail.sysu.edu.cn (J. Z. Liu)

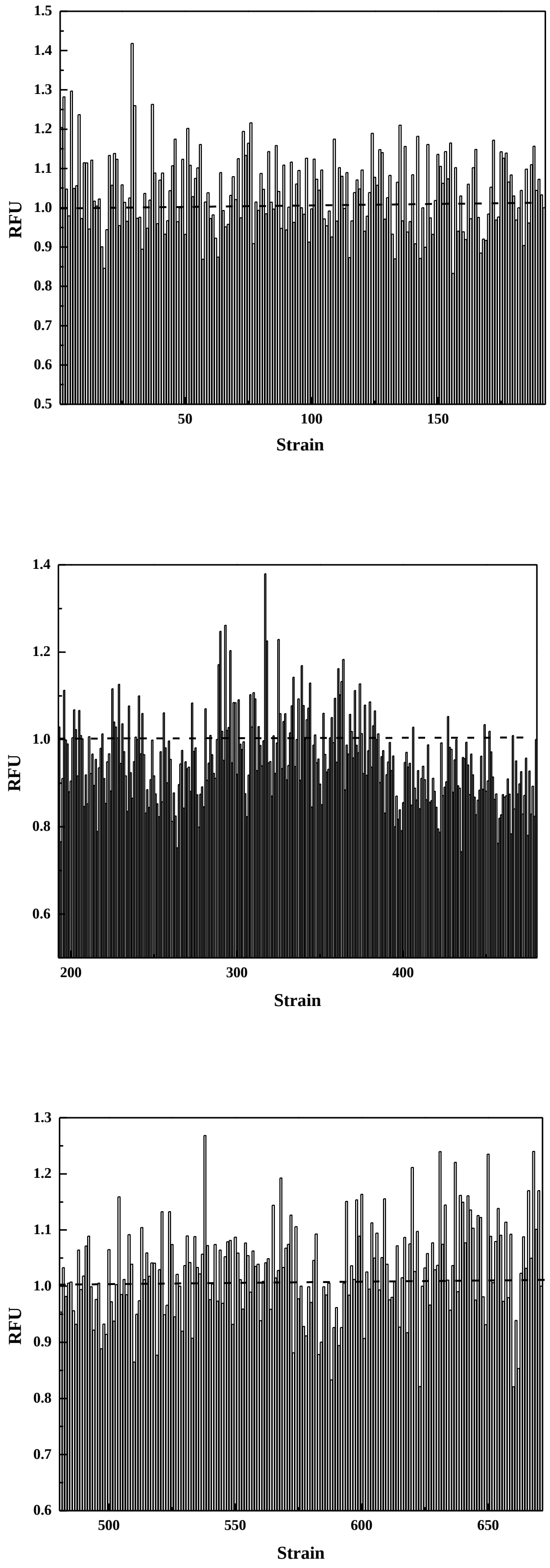

Suppl. Fig. 1 The fluorescent strength of the adaptive laboratory evolution strains

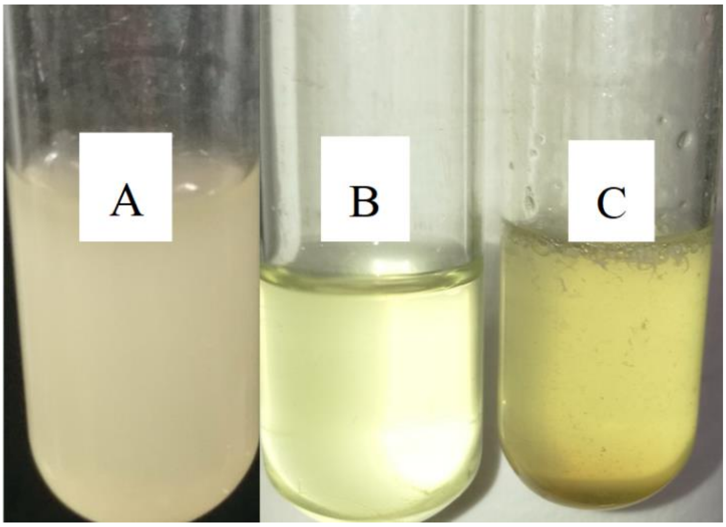

Suppl. Fig. 2 Growth of *E. coli* YZ-3-A-T and its mutants after ARTP mutagenesis. *E. coli* YZ-3-A-T were cultured in the presence of 2% pinene (A) and 35 mM fosmidomycin (B); the mutants were cultured in the presence of 35 mM fosmidomycin and 2% pinene (C)

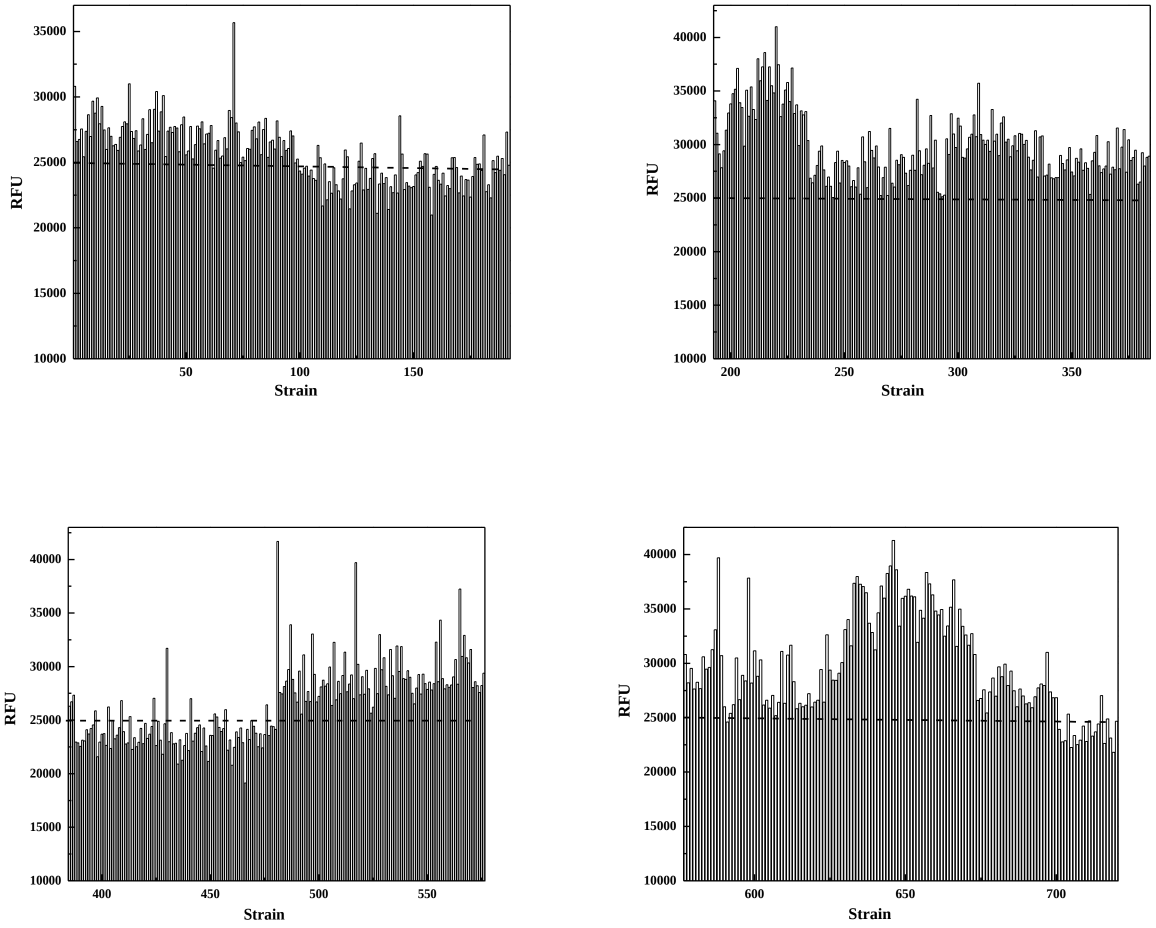

Suppl. Fig. 3 The fluorescent strength of the selected mutant resistant to fosmidomycin

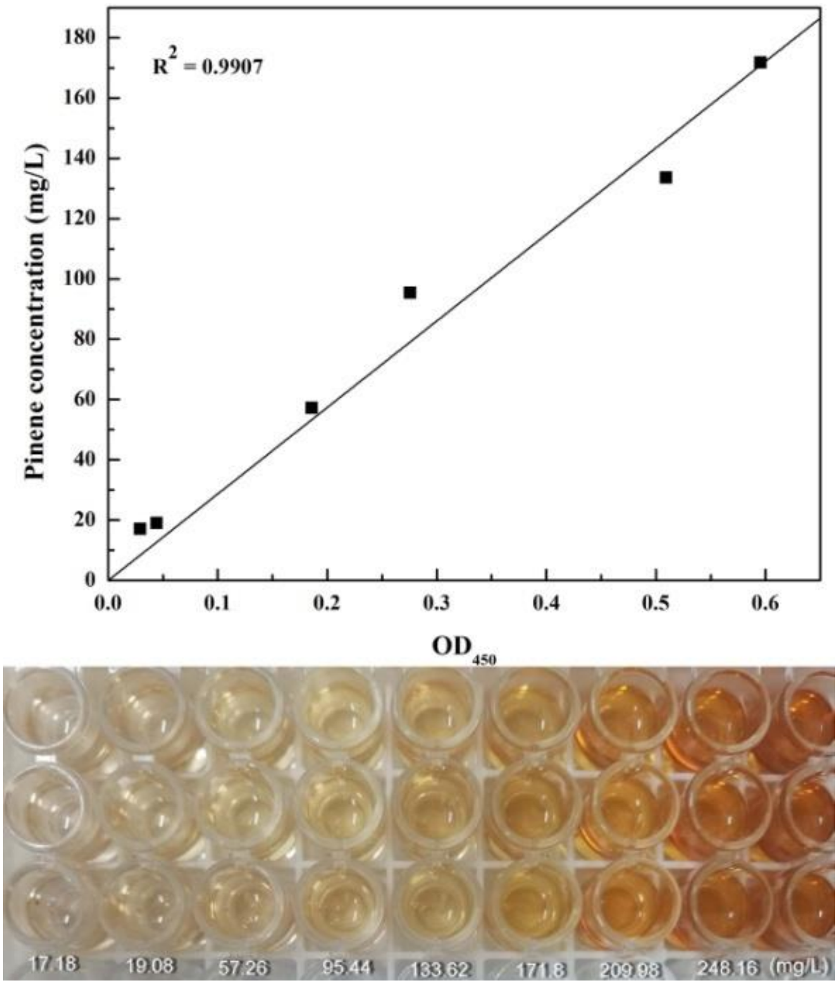

Suppl. Fig. 4 The relationship between the OD_{450}value of the pinene reaction solution with concentrated sulfuric acid and pinene concentration

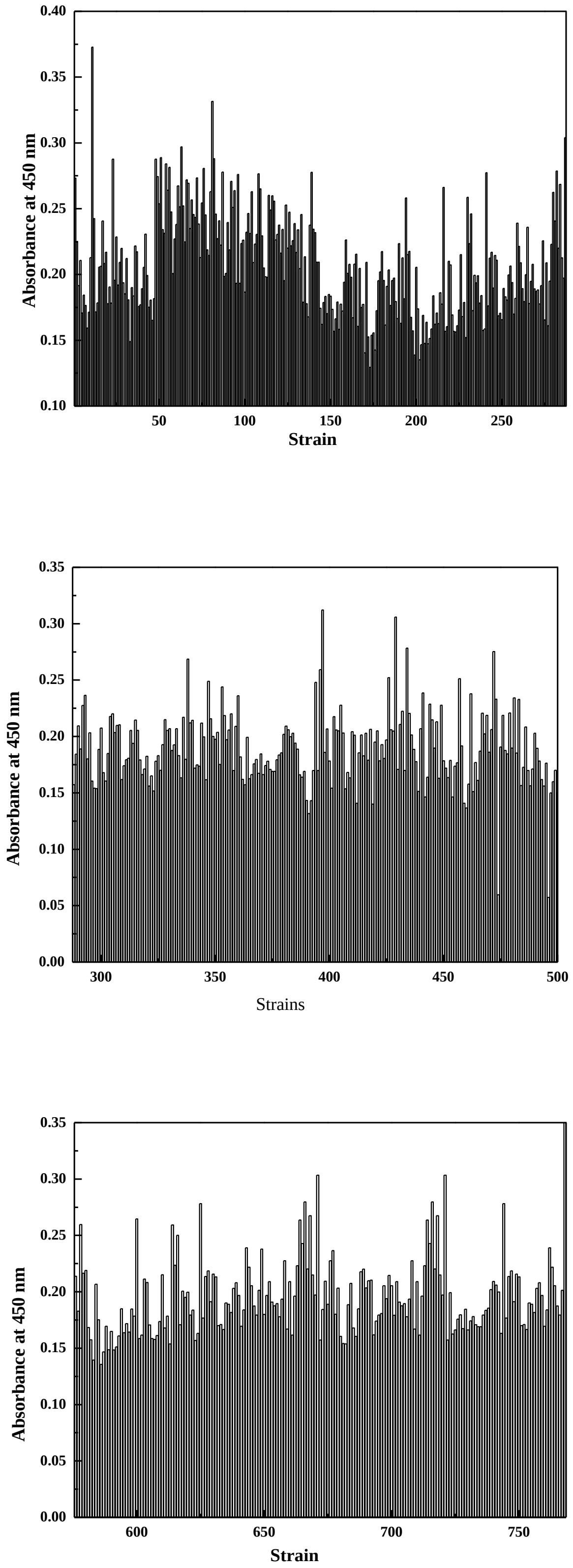

Suppl. Fig. 5 Absorbance at 450 nm of the TIGR libraries

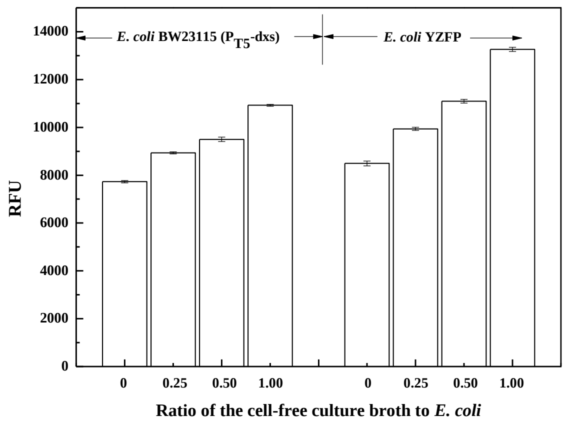

Suppl. Fig. 6 Effect of the cell-free culture broth of *E. coli* MEVI on fluorescence strength in *E. coli*. *E. coli* harboring pP_{rstA}-GFP was incubated at 30°C and 130 rpm until an OD_{600} of 4.0 was reached. Then the cell-free culture broth of *E. coli* MEVI cultured overnight was added. The cultures were incubated for additional 12 h and then the fluorescence strengths were assayed.
